# Spatial and Temporal Mapping of Breast Cancer Lung Metastases Identify TREM2 Macrophages as Regulators of the Metastatic Boundary

**DOI:** 10.1158/2159-8290.CD-23-0299

**Published:** 2023-12-12

**Authors:** Ido Yofe, Tamar Shami, Noam Cohen, Tomer Landsberger, Fadi Sheban, Liat Stoler-Barak, Adam Yalin, Truong San Phan, Baoguo Li, Lea Monteran, Ye’ela Scharff, Amir Giladi, Miriam Elbaz, Eyal David, Anna Gurevich-Shapiro, Chamutal Gur, Ziv Shulman, Neta Erez, Ido Amit

**Affiliations:** 1 Department of Systems Immunology, Weizmann Institute of Science, Rehovot, Israel; 2 Department of Pathology, Sackler Faculty of Medicine, Tel Aviv University, Tel Aviv, Israel

## Abstract

Cancer mortality primarily stems from metastatic recurrence, emphasizing the urgent need for developing effective metastasis-targeted immunotherapies. To better understand the cellular and molecular events shaping metastatic niches, we used a spontaneous breast cancer lung metastasis model to create a single-cell atlas spanning different metastatic stages and regions. We found that premetastatic lungs are infiltrated by inflammatory neutrophils and monocytes, followed by the accumulation of suppressive macrophages with the emergence of metastases. Spatial profiling revealed that metastasis-associated immune cells were present in the metastasis core, with the exception of TREM2^+^ regulatory macrophages uniquely enriched at the metastatic invasive margin, consistent across both murine models and human patient samples. These regulatory macrophages (*Mreg*) contribute to the formation of an immune-suppressive niche, cloaking tumor cells from immune surveillance. Our study provides a compendium of immune cell dynamics across metastatic stages and niches, informing the development of metastasis-targeting immunotherapies.

## Introduction

Breast cancer continues to be one of the leading causes of cancer-related death in women (1, 2). Mortality from breast cancer is almost exclusively a result of tumor metastasis, as advanced metastatic cancers are currently incurable. Lungs are one of the most common sites of breast cancer metastasis, conferring a median survival of less than two years after diagnosis, thus posing a major clinical challenge (3). The early stages of metastasis, between the resection of primary tumor and diagnosis of clinically evident metastasis, are currently a “black box” in human patients, limiting our ability to predict or prevent metastatic relapse. Because these are crucial rate-limiting events, understanding the mechanisms underlying the different stages of the metastatic process, and accurately dissecting specific metastatic niches, is an urgent quest in cancer research, and an essential step toward the discovery of novel therapeutic targets.

Metastasis is a complex multistep process (4, 5). The metastatic microenvironment is crucial in supporting the formation of metastases (6). Nevertheless, as most studies of the microenvironment focused on the primary tumor site, the role of the metastatic tumor microenvironment (mTME) and the molecular cross-talk between immune cells at the metastatic niche that enable metastatic relapse are still poorly characterized.

The recent advancements in single-cell RNA-sequencing (scRNA-seq) technologies have led to improved characterization of immune cell states within the tumor microenvironment (7). ScRNA-seq analyses enabled detailed dissection of myeloid and T-cell heterogeneity across various cancer types (8–10). However, breast cancer studies using this technology have almost exclusively focused on mapping of the immune microenvironment in primary tumors (11), due to the practical and ethical constraints in accessing samples from metastatic lesions, limiting our molecular understanding of the clinically relevant immune milieu, which includes potential metastasis relevant therapeutic targets. Because each microenvironment exerts specific signals that support or oppose colonization and expansion of disseminated tumor cells (12, 13), understanding organ-specific mechanisms that enable metastatic growth is of crucial importance.

A fundamental phase in the metastatic process is the establishment of a premetastatic niche, preceding the formation of clinically relevant metastases (14). Premetastatic preparation of secondary sites to facilitate subsequent tumor cell colonization is mediated by secreted factors from tumor and stromal cells that instigate a permissive premetastatic niche by influencing the recruitment and functional activation of immune cells (15–21). Specifically, macrophages and neutrophils were shown to play important roles in facilitating breast cancer metastasis to the lungs (5, 22, 23), but detailed spatial and temporal mapping of their variations in the premetastatic niche is still lacking.

Tumors are composed of various stromal and immune cell types that nurture and support the colonization and growth of tumor cells. This cellular landscape undergoes alterations as tumors develop and spread. However, a further layer of complication is that cellular heterogeneity is not uniform in the spatial axis. Tumors are composed of distinct cellular niches, and cells do not operate in solitude, but rather maintain intricate cross-talk with other cells in their surroundings (24, 25). Therefore, accurate characterization of specific functional immune subpopulations in the context of defined niches is vital.

To address these unmet needs, we mapped the immune microenvironment in single-cell resolution during distinct stages of breast cancer lung metastasis. In order to isolate metastases-associated immune cells, we utilized spatial NICHE-seq technology (26), applied to mouse models of spontaneous lung metastasis subsequent to resection of the primary breast tumor, to generate a dynamic atlas of immune cell states throughout the metastatic process and across spatial regions. We observed profound differences between the TME of the primary tumor and that of lung metastases. Notably, metastases exhibit tumor-associated monocytes and macrophages of different states. Prior to the formation of metastases, the premetastatic niche is established, characterized by the tissue infiltration of monocytes and neutrophils with inflammatory signatures. We have determined that the onset of metastatic growth is associated with an increase in suppressive monocytes and macrophages, conventional type 2 dendritic cells (cDC2), and regulatory T cells (Treg). In addition, the lung milieu of metastatic regions, yet not that from distal normal tissue, provided immune cell migratory signals. Upon focusing on the core or invasive margins of the metastasis through photo-labeling, we have discovered that glycolytic metabolism and type-1 interferon signaling are dominant features of activated DCs, monocytes, and macrophages in the metastatic core. Conversely, regulatory macrophages expressing *Trem2* with extracellular matrix (ECM) remodeling features and lipid metabolism are spatially distinct, forming a suppressive niche at the invasive margin of the metastases. These findings were validated in different mouse models of breast cancer lung metastasis as well as in human lung metastasis across multiple cancer types. Our comprehensive analyses of the immune microenvironment revealed key alterations in immune cell molecular states during the metastatic cascade and elucidated the diverse landscapes of immune subpopulations in distinct spatial compartments of breast cancer metastases.

## Results

### Detailed Atlas of the Immune Microenvironment in Breast Cancer Lung Metastasis

**Figure 1:**
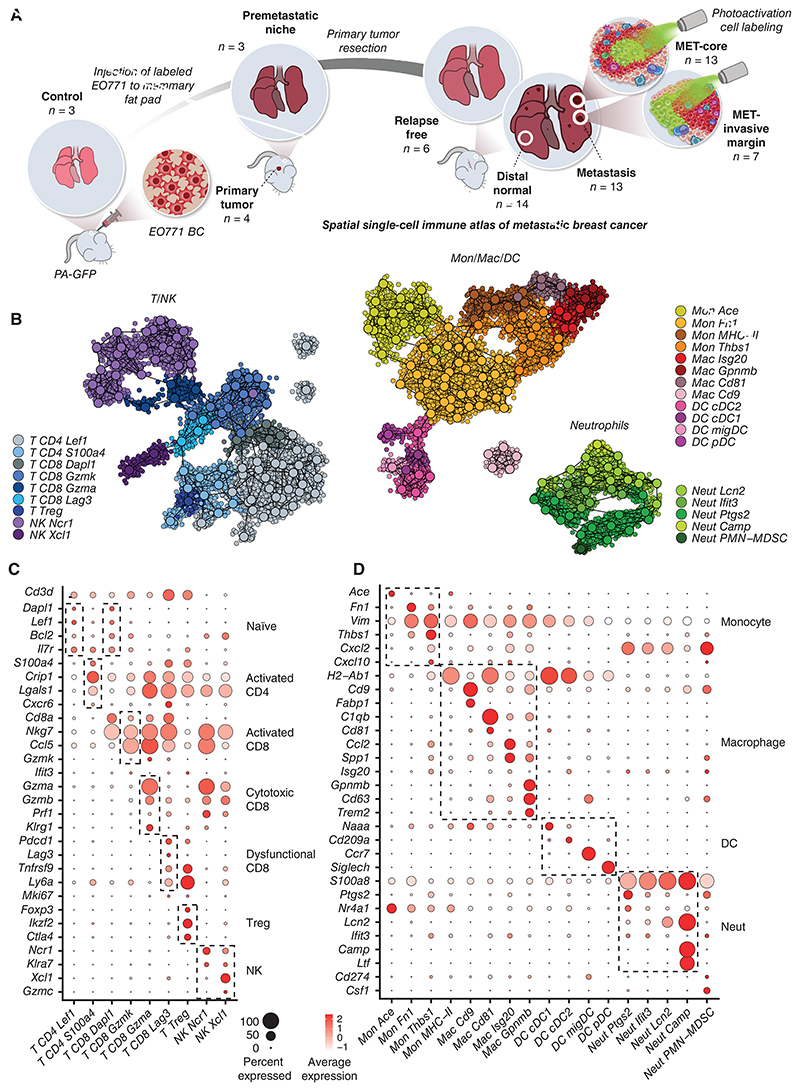
Detailed atlas of the immune microenvironment in breast cancer lung metastasis. **A**, Experimental design of the spontaneous metastasis model conducted in photoactivatable-GFP mice. **B**, Two-dimensional projection of the transcriptomic profiles of cells from three immune subsets: T and NK; monocyte, macrophage, and DC; and neutrophils. Dots represent single cells and are color-coded according to subpopulation annotation, larger circles represent metacells. See Methods. **C**, Bubble heat map showing marker gene expression across T and NK cell types from **B**. Size indicates the fraction of expressing cells. Color indicates the mean log-normalized expression levels. **D**, As in **C** but for myeloid populations.

Recent studies have characterized the immune microenvironment in primary breast tumors using scRNA-seq (27, 28). However, comprehensive analysis of the metastatic immune microenvironment has mostly been overlooked. To define the principles that govern heterogeneity and plasticity in the metastatic microenvironment, as well as in the premetastatic niche, we set out to temporally and spatially dissect the functional subpopulations of metastases-associated immune cells during metastatic progression. To this end, we utilized a mouse model of spontaneous lung metastasis by orthotopically injecting tdTomato-EO771 breast cancer cells to mammary glands (ref. 29; Fig. 1A). To mimic the clinical setting, primary tumors were surgically resected and spontaneous lung metastases ensued (Supplementary Fig. S1A).

We isolated and analyzed immune cells at different stages and spatial regions. We collected cells from the premetastatic niches (pre-MET) in mice bearing primary breast tumors prior to resection (30), and from mice bearing evident metastatic lesions. Then, following primary tumors resection, mice were monitored for metastasis weekly using CT, and samples were collected shortly after the first CT detection of metastasis. Using tdTomato labeling and microscopic inspection, we were able to distinguish between lung tissues containing metastatic lesions and distal normal tissue. Cells were also collected from mice that did not develop metastases, classified as relapse-free.

To dissect and compare the immune microenvironment in different spatial regions, we utilized photoactivatable GFP (PA-GFP) mice (26) as recipients of breast tumors and emergent spontaneous metastasis, thus enabling spatial analysis by application of the NICHE-seq technology. The NICHE-seq methodology enables spatial analysis of small and irregular niches as it combines photoactivatable fluorescent reporters, coupled to two-photon microscopy and massively parallel single-cell RNA-seq (26). NICHE-seq profiling is based on sorting and analysis from within visually selected metastatic microenvironments in PA-GFP transgenic mice, where tumor cells are fluorescently labeled, and photoactivated host cells are labeled through PA-GFP photoactivation (Fig. 1A). The NICHE-seq metastasis experimental system enables dissection of micro-niches (as small as ~1,000 cells) from fresh, unfixed tissues that are otherwise pathologically indistinguishable from the adjacent healthy tissue. We performed photoactivation on metastases-bearing lungs, labeling either the metastasis core or the metastasis invasive margin. Finally, we isolated immune cells from the lungs of control PA-GFP mice and also included in our atlas immune cells from EO771-tdTomato primary tumors. Cells from all samples were isolated by FACS, gating on CD31^−^CD45^+^ immune cells or CD31^−^GFP^+^ cells following photoactivation of metastatic tissues (Supplementary Fig. S1B). ScRNA-seq was performed on 63 samples from 27 mice. After filtering, 24,020 immune cells were retained for subsequent analysis (Supplementary Fig. S1C). Unsupervised clustering divided the immune cells into transcriptionally distinct populations (ref. 31; Supplementary Tables S1 and S2).

Using marker genes and coexpressed gene modules, we annotated immune cell populations to find all immune cell lineages (Supplementary Fig. S1D and S1E). We identified nine populations of T and natural killer (NK) cells (Fig. 1B and C), including naïve CD4 and CD8 cells (*CD4 Lel1* and *CD8 Dapl1*), activated CD4 (*CD4 S100a4*) and CD8 (*CD8 Gzmk* and *CD8 Gzma*) cells, and dysfunctional CD8 (*CD8 Lag3*) and *Treg* (*Foxp3*) cells. NKs were divided into two populations, *NK Ncr1* and *NK Xcl1*. The myeloid cells consisted of dendritic cells (DC), monocytes, macrophages, and neutrophils (Fig. 1D). Dendritic cells were partitioned into *cDC1* (Naaa), *cDC2* (*CD209a*), migratory DC (*migDC, Ccr7*), and plasmacytoid (*Siglech*). Monocytes were segregated into classic monocytes (*Mon Ace*), and monocyte subsets expressing fibronectin (*Mon Fn1*) or Thrombospondin (*Mon Thbs1*). Macrophages were divided into alveolar (*Mac Cd9*), tumor-associated macrophages (*Mac Cd81* and *Mac Isg20*), and a macrophage population that we have previously identified as macrophage regulatory cells (*Mregs)*, uniquely expressing genes such as *Trem2, Gpnmb*, and *Cd63* (32). Neutrophils, identified by *S100a8* expression, were divided into subsets by expression of *Ptgs2* (coding for prostaglandin-endoperoxide synthase, or COX2), *Ifit3, Lcn2*, and *Camp*. In addition, a neutrophil-associated population of polymorphonuclear myeloid-derived suppressor cells (*PMN-MDSC*) was identified, expressing *Csf1* and *Cd274* (PD-L1; Supplementary Tables S3 and S4).

### Lung Metastases and Primary Tumors Exhibit Divergent Immune Landscapes

**Figure 2:**
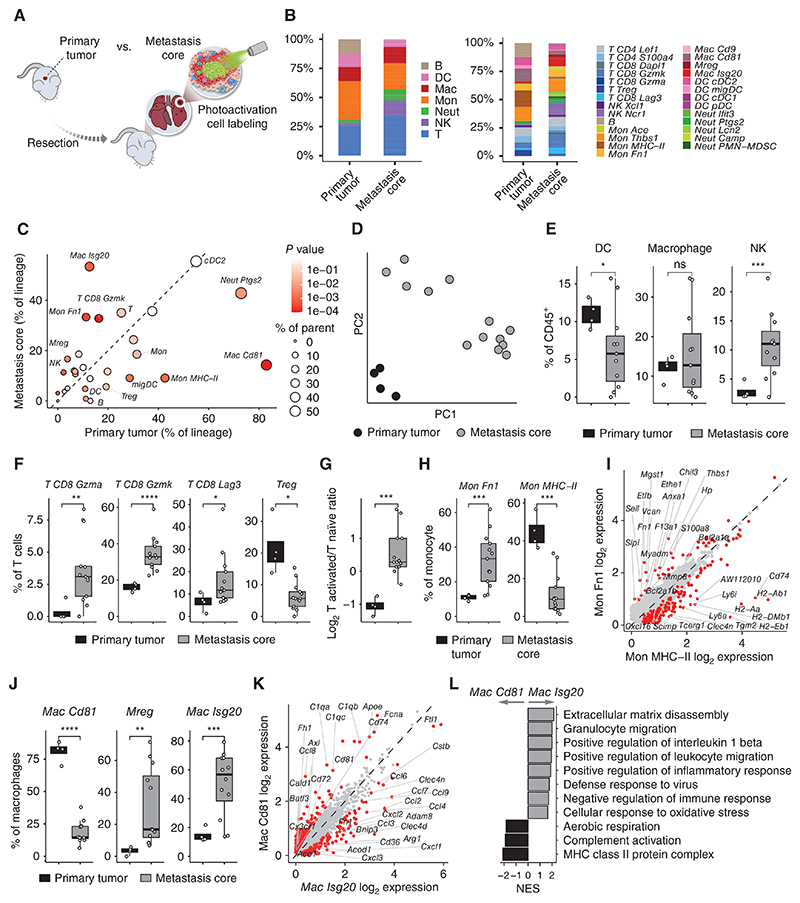
Lung metastases and primary tumors exhibit divergent immune landscapes. **A**, Scheme illustrating the comparison of primary breast tumors and lung metastases. **B**, The cumulative cell fraction of the different main immune lineages (left) and subtypes (right) in primary tumors (*n* = 4 samples) and metastasis core (*n* = 13). **C**, Fractions of cells belonging to different immune lineages (from total) or subtypes (from their respective lineage), averaged over primary tumor (*x*-axis) or metastasis core (*y*-axis) samples. Size indicates the average of x and y. Color depicts *P* value of the two-sided T test between x and y, accounting for sample variation. **D**, PCA of immune compartment makeup, based on cell type and subpopulation fractions. **E**, Fractions of indicated cell types out of total CD45^+^ cells. **F**, Fractions of indicated T-cell subtypes from total T cells. **G**, The log_2_ ratio of activated T cells (*Cd8 Gzma* and *Cd8 Gzmk*) and naïve T cells (*Cd8 Dapl1* and *Cd4 Lef1*). **H**, Fractions of the indicated monocyte subtypes from total monocytes. **I**, Comparison of monocyte subtypes gene expression (log_2_ normalized). **J**, Fractions of the indicated macrophage subtypes from the total macrophage. **K**, Comparison of macrophage subtypes gene expression (log_2_ normalized). **L**, Enriched gene ontology terms in macrophage subtypes. Two-tailed Student *t* test was used. In box plots, the center line represents the median, the box limits denote the 25th to the 75th percentiles, and the whiskers represent the minimum and maximum values. Differentially expressed genes (DEG) are colored in red and leading DEGs are labeled. Normalized gene ontology term enrichment score (NES) is shown on the *x*-axis. For all terms, *P*_adj_ < 0.05.

Countless studies use murine models of orthotopically implanted breast cancer cells to investigate the TME in primary tumor growth, for preclinical drug testing (including that of immunotherapies) and other applications. However, the transition from such models into human settings is under continual debate, as many drugs discovered in murine models fail in the clinic. One of the reasons for this gap in translation may be the different characteristics and immune milieu of the metastatic sites. Therefore, we initially mapped the differences between the microenvironment of the primary site and that of the metastatic niche, to find targetable nodes for metastasis treatment that would be missed had we only considered primary tumors. We used NICHE-seq to label specifically metastasis residing cells in the spontaneous metastasis model (Fig. 2A), comparing immune cells from both primary breast tumors and lung metastatic core, rather than averaging whole lung tissue (26). To ensure that photoactivation of lung tissues does not elicit a bias in the cells captured, we performed photoactivation on control mice lung tissues and sorted CD31^−^CD45^+^ cells that were either positive or negative for GFP. Our analysis verified that no significant changes in the frequency of cell populations and gene expression resulted from photoactivation (Supplementary Fig. S2A and S2B).

We compared the overall immune composition between metastases and primary tumors (PT) at the cell type and subpopulation level and found that the immune landscape of primary tumors and the metastasis core are highly divergent (Fig. 2B and C). Principal component analysis (PCA) based on cell type and subpopulation frequencies revealed a clear separation between the two locations (Fig. 2D), indicating that the immune landscape of PT is drastically different from that found in metastases. Specifically, examining the differences in main immune lineages, we found that NK cells are increased, whereas B and DC are reduced in metastasis compared with the primary tumor (Fig. 2E; Supplementary Fig. S2C). Although the total proportion of T cells, monocytes and macrophages did not differ between PT and metastasis, we found differences in subpopulation composition (Supplementary Fig. S2D–S2G). Although the primary tumor is associated with increased levels of Tregs, metastasis had increased levels of activated *CD8 Gzmk* cells, *CD8 Gzma*, the dysfunctional *CD8 Lag3* T cells, and an overall increase in activated/naïve T-cell ratio (Fig. 2F and G), indicating that a distinct set of signals shape the metastatic microenvironment. The major monocyte population in both metastasis and primary tumor was *Mon Thbs1*; however, metastasis had increased *Mon Fn1* fraction and reduction in antigen-presenting *Mon MHC-II* (Fig 2H and I). Moreover, although metastases were infiltrated by *Mac Isg20* and *Mregs*, macrophages in the primary tumor were mostly of the *Mac Cd81* population (Fig. 2J), expressing elevated levels of complement system genes (*C1qa/b/c, Fcna*), MHC-II (*H2-Ab1, Cd74, Cd81*), and *Ccl8*, which has been demonstrated as enriched in breast cancer tumor-associated macrophages (TAM), and supporting cancer cell dissemination (Fig. 2K and L; ref. 33). Thus, the metastatic immune microenvironment is composed of distinct cell subsets, pathways, and checkpoints, and these differences should be carefully considered when designing studies for preclinical drug development.

### The Premetastatic Lung Microenvironment Is Characterized by Activation of Monocytes and Neutrophils

**Figure 3:**
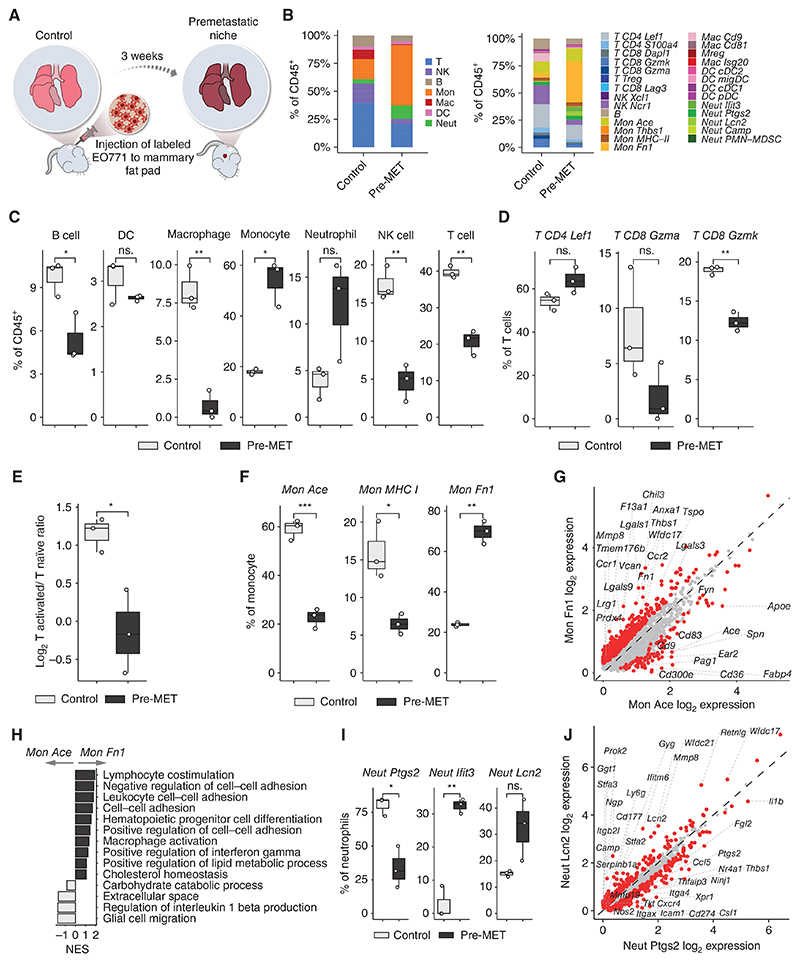

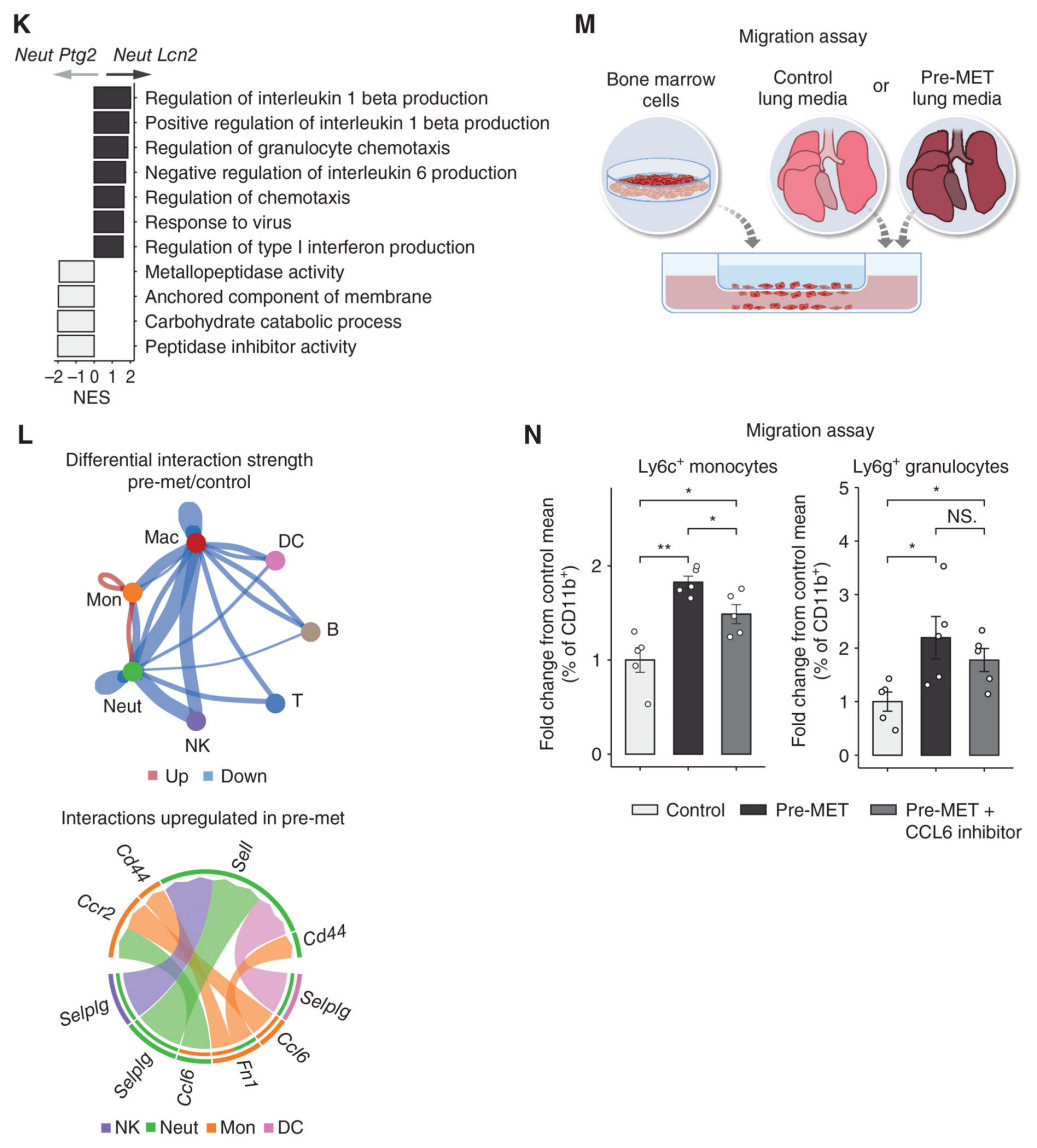
The premetastatic lung microenvironment is characterized by the activation of monocytes and neutrophils. **A**, Scheme illustrating the comparison of control and premetastatic lung tissues. **B**, The cumulative fraction of the different main immune lineages (left) and subtypes (right) in control (*n* = 3 samples) and premetastatic (Pre-MET, *n* = 3) samples. **C**, Fractions of indicated cell types out of total CD45^+^ cells. **D**, Fractions of indicated T-cell subtypes from total T cells. **E**, The log_2_ ratio of activated T cells (*Cd8 Gzma* and *Cd8 Gzmk*) and naïve T cells (*Cd8 Dapl1* and *Cd4 Lef1*). **F**, Fractions of the indicated monocyte subtypes from total monocytes. **G**, Comparison of monocyte subtypes gene expression (log_2_ normalized). **H**, Enriched gene ontology terms in monocyte subtypes. **I**, Fractions of the indicated neutrophil subtypes from total monocytes (ns. = non significant). **J**, Comparison of neutrophil subtypes gene expression (log_2_ normalized). **K**, Enriched gene ontology terms in neutrophil subtypes. **L**, CellChat analysis (see Methods) of differential interaction strength between cell types in Pre-MET and control lungs based on ligand–receptor gene expression (top), and of upregulated ligand–receptor interactions in Pre-MET cells (bottom). Up depicts higher in Pre-MET. **M**, Scheme illustrating *ex vivo* cell migration assay. Cells were purified from the bone marrow of normal mice. The lung noncellular fraction was produced from the lungs of control or Pre-MET mice, and the migration of monocytes and granulocytes toward lung-secreted factors was analyzed. **N**, Quantification of migrated Ly6c^+^ monocytes and Ly6g^+^ granulocytes toward supernatant from normal or pre-MET lung-secreted factors, with or without anti-CCL6 antibody (presented as fold change from the normal mean; error bars, SE). Two-tailed Student *t* test was used. In box plots, the center line represents the median, the box limits denote the 25th to the 75th percentiles, and the whiskers represent the minimum and maximum values. Differentially expressed genes (DEG) are colored in red and leading DEGs are labeled. Normalized gene ontology term enrichment score (NES) is shown on the *x*-axis. For all terms, *P*_adj_ < 0.05.

Motivated by these insights, we set out to comprehensibly characterize the lung metastasis TME (mTME). Premetastatic formation of secondary sites that facilitate subsequent tumor cell colonization is recognized to be an important stage in the metastatic cascade (6). To investigate the changes that occur in the lung immune landscape prior to metastasis onset, we compared the immune milieu of normal lungs with that of lungs from tumor-bearing mice on day 20 after EO771-tdTomato cell line injection, before resection of PTs (Fig. 3A). Premetastatic lungs (Pre-MET) were defined by lack of metastatic lesions by microscopic inspection, CT imaging, and detection of tdTomato^+^ cells by FACS analysis (Supplementary Fig. S3A). We found that tumor-bearing mice had significant modifications of the lung immune milieu already at this premetastatic stage (Fig. 3B; Supplementary Fig. S3B). Specifically, macrophages, B, NK, and T cells, were reduced, while neutrophils and monocytes in the Pre-MET niche were increased (Fig. 3C). Interestingly, the proportion of resident (alveolar) macrophages was drastically diminished in the Pre-MET microenvironment (Fig. 3C). These findings are in line with previous studies that reported expansion of monocytes and neutrophils at the premetastatic stage of lung metastasis (34, 35). Moreover, the neutrophils to lymphocytes ratio (NLR) was increased in Pre-MET lungs in EO771-injected mice (Supplementary Fig. S3C). To ensure that our findings are not model specific or mice strain specific, we performed similar experiments in an additional mouse model of triple-negative breast cancer. We orthotopically injected WT (Balb/c) mice with a 4T1-tdTomato cell line, surgically removed PT after 3 weeks, and performed CT monitoring weekly to follow-up on spontaneous lung metastatic relapse. Notably, a similar increase in neutrophils and decrease in T cells was also evident in Pre-MET lungs of mice injected with the 4T1 breast cancer model (Supplementary Fig. S3D), suggesting that this mechanism is generally important in shaping the lung metastatic niche. NLR is clinically used as a prognostic biomarker in several cancer indications, and high blood NLR is correlated with poor outcomes (18). Our findings suggest that these changes are instigated early during the metastatic cascade and are already operative at premetastatic stages.

In addition to the overall reduction of lymphocytes, we observed a 2-fold reduction of activated *CD8 Gzmk* and cytotoxic *CD8 Gzma* T cells, expressing granzymes (*Gzma, Gzmb, Gzmk*), chemoattractants (*Ccl4, Ccl5*), and killer cell family genes (*Klrc1, Klrc2*; Fig. 3D and E). These findings are in line with a previous study that reported an increase in T-cell dysfunction in premetastatic lungs (35). These early immune alterations, possibly instigated by systemic signaling from the primary tumor, implicate immune suppression in the formation of the premetastatic niche, possibly enabling immune evasion and thriving of disseminated cancer cells that arrive at the niche.

The most abundant immune cells in Pre-MET lungs are monocytes, which also exhibited the highest increase compared with control (Fig. 3B and C). This pronounced change coincided with a switch in monocyte composition, wherein classic monocytes (*Mon Ace*) are replaced by the *Mon Fn1* population in Pre-MET (Fig. 3F). The *Mon Fn1* cell population is characterized by inflammatory features, including increased expression of the chemokine receptors *Ccr2* and *Ccr1* facilitating cell recruitment, immune suppression and tumor-promoting factors such as galectins (*Lgals3/9*), and the NLRP3 inflammasome inhibitor *Tmem176b* (Fig. 3G). Moreover, compared with the classic monocytes *Mon Ace* that populate healthy lungs, *Mon Fn1* cells are enriched for ECM-associated factors such as *Mmp8, Vcan*, and fibronectin (*Fn1*), and cell–cell adhesion and lipid metabolic processes (such as low-density lipoprotein receptor *Ldlr*). These molecular profiles may suggest that ECM remodeling and metabolic alterations precede metastatic onset (Fig. 3H; Supplementary Fig. S3E).

Similar to monocytes, the neutrophil composition switched from mature cells with high prostaglandin expression (*Neut Ptgs2)*, to proinflammatory cells (*Neut Ifit3* and *Neut Lcn2)* in Pre-Met lung niches (Fig. 3I). Inflammatory neutrophils upregulated the expression of IFN-I signaling, of neutrophil secondary granule factors (*Ngp*), and of *Padi4*, required for neutrophil extracellular trap (NET) formation (Fig. 3J; Supplementary Fig. S3F; ref. 36). Gene set enrichment analysis (GSEA) revealed the Pre-MET—enriched *Neut Lcn2* to be high in chemotactic pathways, IL1β production, and IFN-I, compared with *Neut Ptgs2* found in normal lungs (Fig. 3K). These findings suggest that neutrophils mediate an inflammatory microenvironment in the premetastatic niche, promoting metastatic progression.

To assess the interactions between the different immune populations, we next analyzed the cell-to-cell interactions within the immune compartments of Pre-MET and control lungs, using the CellChat algorithm (37). We found an overall reduction in interaction strength within Pre-Met lungs, except for monocyte–neutrophil interactions, and monocyte–autocrine interactions (Fig. 3L). Specifically, interactions that were upregulated in premetastatic lungs included the Ccl6 (monocyte)–Ccr2 (neutrophil) signaling axis (Fig. 3L; Supplementary Fig. S3G). We therefore hypothesized that these secreted factors may be involved in the recruitment of monocytes and neutrophils to premetastatic lungs. To test this, we performed an *ex-vivo* transwell migration assay. Lung tissues from control and premetastatic mice were collected and processed into single-cell suspensions and the noncellular fraction of this lung homogenate was used as chemoattraction media for bone marrow–derived cells (Fig. 3M). The migration of bone marrow cells was analyzed by flow cytometry. We found that secreted factors from Pre-MET lungs significantly enhanced monocyte (CD45^+^Ly6C^+^) and granulocyte (CD45^+^Ly6G^+^) migration, compared with secreted factors from normal lungs (Fig. 3N; Supplementary Fig. S3H). Moreover, functional inhibition of CCL6 in the Pre-MET lung homogenate significantly reduced monocyte recruitment (Fig. 3N), suggesting a functional role for this signaling axis in the shaping of the immune metastatic niche.

In summary, these data reveal that in the presence of a primary breast cancer tumor, the lung immune milieu undergoes vast remodeling, including an influx of specific inflammatory populations of monocytes and neutrophils, whereas alveolar macrophages, activated T cells, and NK cell populations are diminished. These systemic changes may contribute to the formation of a hospitable microenvironment, conducive to the seeding and expansion of disseminated cancer cells.

### Distinct Immune Populations Define Metastasis Spatial Niches

**Figure 4:**
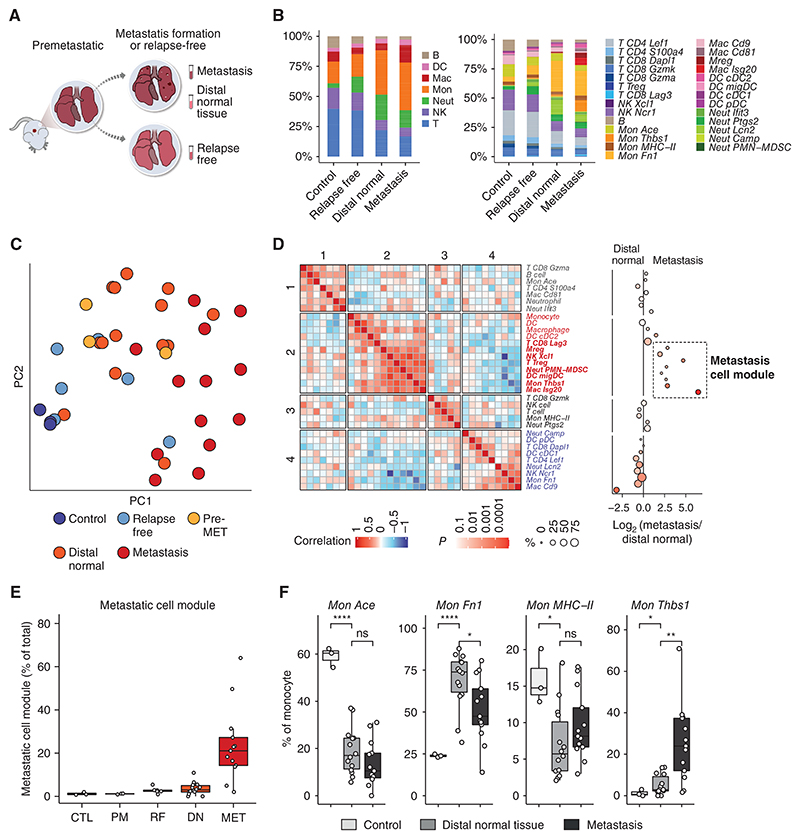

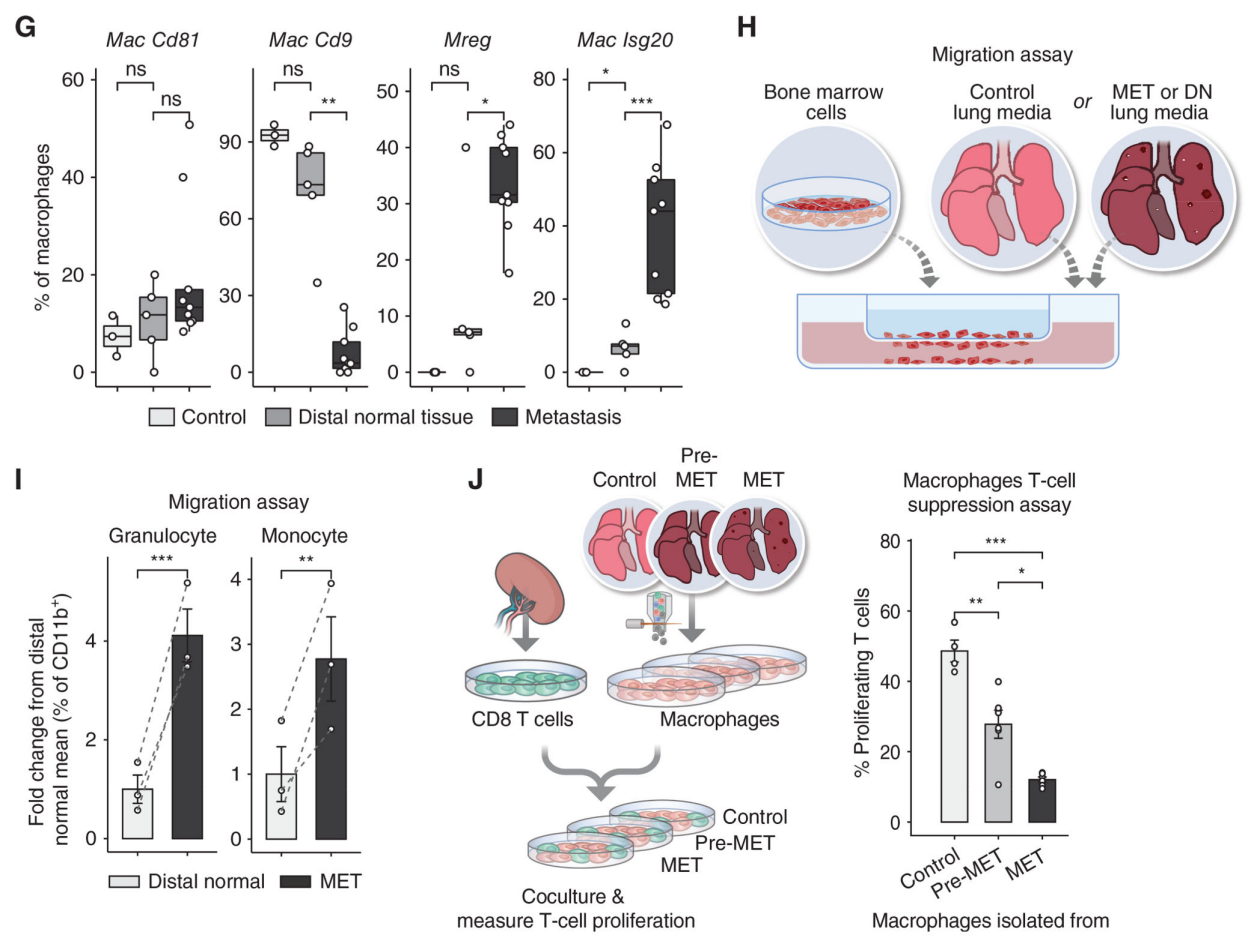
Progression to lung metastasis is associated with infiltration by unconventional immune cell subtypes. **A**, Scheme illustrating the comparison of control, relapse-free, distal normal, and metastasis lung tissues. **B**, The cumulative fraction of the different main immune cell lineages (left) and subtypes (right) per tissue type (control, *n* = 3 samples; relapse free, *n* = 6; distal normal, *n* = 14; metastasis, *n* = 13). **C**, PCA of immune compartment makeup, based on cell type and subtype fractions. **D**, Cellular module analysis. Pairwise Spearman correlation of cell type and subpopulation fraction across samples of distal normal and metastasis (left; color gradient represents Spearman correlation). Consensus hierarchical clustering into four cell modules. Enrichment of each cell type between distal normal and metastasis tissues (right). Size indicates the mean percentage of cells in all samples; color gradient represents the *P* value of Student *t* test between metastasis and distal normal per cell population. **E**, Quantification of cell fractions from total cells per sample in the metastasis cell module analysis in **D**. CTL, control; PM, premetastasis; RF, relapse free; DN, distal normal; MET, metastasis. **F**, Fractions of the indicated monocyte subtypes from total monocytes. **G**, Fractions of the indicated macrophage subtypes from total macrophages. **H**, Scheme illustrating *ex vivo* cell migration assay. Cells were purified from the bone marrow of normal mice. The lung noncellular fraction was produced from the distal normal area or metastatic area of metastases-bearing lungs. Migration of monocytes and granulocytes toward lung-secreted factors was analyzed. **I**, Quantification of migrated Ly6c^+^ monocytes and Ly6g^+^ granulocytes toward supernatant from the distal normal area or metastatic area of metastases-bearing lungs (presented as log_2_ fold change from the distal normal mean; error bars, SE. Two-tailed paired Student *t* test was used). **J**, Scheme and quantification of macrophage-induced T-cell suppression assay. Splenic T cells from normal mice were stimulated and stained with cell proliferation dye and then cocultured for 48 hours with macrophages isolated from the lungs of control, premetastatic (Pre-MET), or metastasis-bearing mice. Error bars, SE. Two-tailed Student *t* test was used. In box plots, the center line represents the median, the box limits denote the 25th to the 75th percentiles, and the whiskers represent the minimum and maximum values.

To better understand the composition of the metastatic niche, we next characterized the immune mTME niche itself and analyzed cells from lung metastatic lesions compared with distal normal tissues of the same lung, and with control mice (Fig. 4A). Similar to the immune remodeling that we identified in pre-MET lungs, both distal normal and metastasis tissues exhibited an enrichment of monocytes and neutrophils, and conversely a lower abundance of B cells, lymphocytes, and NKs, compared with control, representing persistence of the Pre-MET phenotype (Fig. 4B). Interestingly, the immune microenvironment in lungs of mice that never developed lung metastases (relapse free) was similar to that of control mice (Fig. 4B; Supplementary Fig. S4A), further suggesting that reshaping of the immune mTME is essential for metastatic progression.

Analysis of the differences in the main immune lineages between metastasis and distal normal tissues highlighted the enrichment of macrophages as the most significant change (Fig. 4B; Supplementary Fig. S4B). To better characterize the differences between spatial niches in lung metastasis, we compared immune subpopulation frequencies and performed PCA of cellular compositions at different spatial regions, which identified three tissue archetypes (Fig. 4C). One consisting of tissues from nontumor/metastasis-bearing mice, namely, control samples and relapse-free sites, rich in lymphocytes and NK cells (blue). The second consisted of Pre-MET and distal normal tissues, rich in neutrophils and monocytes, specifically *Mon Fn1* (orange). The third archetype consisted of metastasis tissues, rich in IFN-I–expressing monocytes, macrophages, and suppressive *Mregs* (red).

To further identify cellular modules separating metastasis and distal normal tissues, we correlated the tissue composition of the different samples (Fig. 4D). Consensus hierarchical clustering revealed four cell modules. Specifically, we identified a suppressive cell module, consisting of dysfunctional *CD8 Lag3, Tregs, PMN-MDSCs, Mregs*, and the IFN-I expressing *Mon Thbs1* and *Mac Isg20* subpopulations. This cell module was exclusively found in metastasis (Fig. 4E; Supplementary Fig. S4C–S4E).

Monocytes were the most abundant cell type in distal normal and metastasis tissue (as in Pre-MET), showing the highest increase compared with control (Fig. 4B; Supplementary Fig. S4B). This infiltration consisted of specific monocyte subsets. Both distal normal and metastasis tissues are relatively devoid of the classic circulating monocyte phenotype (*Mon Ace*), replaced by the *Mon Fn1* population (the main constituent of Pre-MET lungs). Metastasis tissues were also highly infiltrated by the *Mon Thbs1* population, and distal normal to a lesser extent (Fig. 4F). Interestingly, *Mon Thbs1* upregulated migration-inducing chemokines (*Ccl2, Ccl7, Ccl12*, and *Cxcl16*), glucose catabolism genes (*Aldoa* and *Eno1*), vasculature development genes (*Vegfa, Ptgs2/COX2, Thbs1*), and IFN-I signaling genes (*Irf7, Ifit3, Rsad2, Cxcl10*) compared with the *Mon Fn1* (Supplementary Fig. S4F and S4G). This gene-expression profile may indicate that metastasis-specific monocytes support processes of inflammation and vessel formation in the initial metastatic lesion.

Macrophages in metastases were both expanded and phenotypically distinct, with two specific populations of metastasis-associated macrophages, *Mac Isg20* and *Mreg* (Fig. 4G). Comparing macrophages from metastasis and distal normal tissues, we found that metastasis-associated macrophages are characterized by increased expression of genes related to IL1β production, IFN-I signaling (*Isg20* and *Rsad2*), and genes of immune-suppressive phenotypes such as *Arginase 1*, whereas macrophages from distal normal tissues expressed lipid catabolism programs and tissue-resident genes such as *Car4, Chil3*, and *Ear1* (Supplementary Fig. S4H and S4I). Flow cytometry analysis using a calibrated panel for the different myeloid populations showed that TREM2^+^ macrophages were specific to metastatic tissue in both EO771 and 4T1 breast cancer metastasis models (Supplementary Fig. S4J). Finally, pseudotime analysis applied to the monocyte–macrophage linages with classic monocytes serving as the base point delineated a trajectory where the Pre-MET enriched *Mon Fn1* evolve from them, the metastasis enriched and transcriptionally related *Mon Thbs1* and *Mac Isg20* follow them, with *Mregs* as the latest emerging population (Supplementary Fig. S4K and S4L).

Postulating that the cross-talk between the different immune cell populations in the mTME may contribute to metastatic progression, we next sought to understand the intercellular communication changes that occur in the metastasis immune compartment. Ligand–receptor analysis comparing distal normal and metastasis tissues revealed an increase in interactions between macrophages and monocytes, neutrophils and DCs, and between DCs and monocytes and neutrophils (Supplementary Fig. S5A). Specifically, we found elevated signaling between macrophage-secreted *Ccl2/Ccl7* to *Ccr2* on monocytes and DCs, in metastasis compared with distal normal tissue (Supplementary Fig. S5B and S5C). The *Ccl2–Ccr2* signaling axis is known to have a key role in tumor and metastasis progression and was tested as a therapeutic target in clinical trials (17). In addition, we found increased signaling to neutrophils with elevated levels of *Ccr1*, via macrophages and monocytes expressing *Ccl9* (the mouse homolog of human *CCL15*), and through upregulation of *Ccl5* by *CD8 Gmza* cells. The *Ccl5* cognate receptor, *Ccr5*, was uniquely expressed in the metastasis-enriched populations *Mac Isg20* and *Mon Thbs1* (Supplementary Fig. S4F and S4I; Supplementary Table S1). Furthermore, *Mif–CD44/CD74* and *Spp1–CD44* interactions were elevated in metastasis macrophages and DCs. These signaling axes have been implicated in tumor progression and the support of immune-suppressive macrophage polarization (38, 39) and antitumor immunity (40). Interestingly, the *Cxcl2–Cxcr2* signaling axis (41) was upregulated in distal normal tissues, suggesting that this pathway contributes to immune suppression in distal areas of metastases-bearing lungs that do not contain metastatic lesions. This self-signaling axis is a determinant of neutrophil aging (42). This finding, along with the reduced *Cxcr4*, indicates a less mature neutrophil state in metastatic tissues that could be a result of either the microenvironment or interconnectivity in cells between the two regions.

To further study the functional significance of these signaling pathway changes and the enhancement of chemoattraction signatures, we examined the capacity of the lung metastatic microenvironment to recruit immune cells from the bone marrow. We performed an *ex vivo* experiment to assess the effect of metastasis-secreted factors versus control lungs on bone marrow–derived cells. We found that secreted factors from metastasis-bearing lungs significantly increased the migration of monocytes compared with control lungs (Fig. 4H; Supplementary Fig. S5D). Next, in order to further dissect the spatial source of chemoattraction within metastases-bearing lungs, we performed the assay following dissection of metastatic lesions from lung tissues (MET) and lung tissue from the same mice that did not contain metastatic lesions (distal normal). This division demonstrated that the migration of both monocytes and neutrophils is specifically increased by secreted factors from the metastatic lesions, but not from normal tissues within metastasis-bearing lungs (Fig. 4I). Thus, secreted factors in the metastatic tissue can further attract myeloid cells to the mTME, enhancing the proinflammatory landscape.

Based on their gene expression, we hypothesized that metastases-associated macrophages mediate immune suppression, thus enabling immune evasion. To functionally test this hypothesis, we performed an *ex vivo* T-cell suppression assay. We isolated macrophages (CD45^+^F4/80^+^) from control, Pre-MET, or metastatic lungs by flow cytometry, and cocultured them with T cells (isolated from a naïve mouse). Analysis of T-cell proliferative capacity revealed that macrophages isolated from metastatic lung tissues attenuated the proliferation of T cells compared with macrophages isolated from control or Pre-MET tissues (Fig. 4J; Supplementary Fig. S5E).

In summary, the dramatic reprograming of the metastatic immune microenvironment compared with control lung tissue suggests that metastatic recurrence affects the entire organ. Furthermore, this process is characterized by alterations that are specific to certain regions, as well as the development of micro-niches that promote immune suppression within the metastatic lung tissue.

### The Metastatic Invasive Margin Is Characterized by Suppressive TREM2 Macrophages

**Figure 5:**
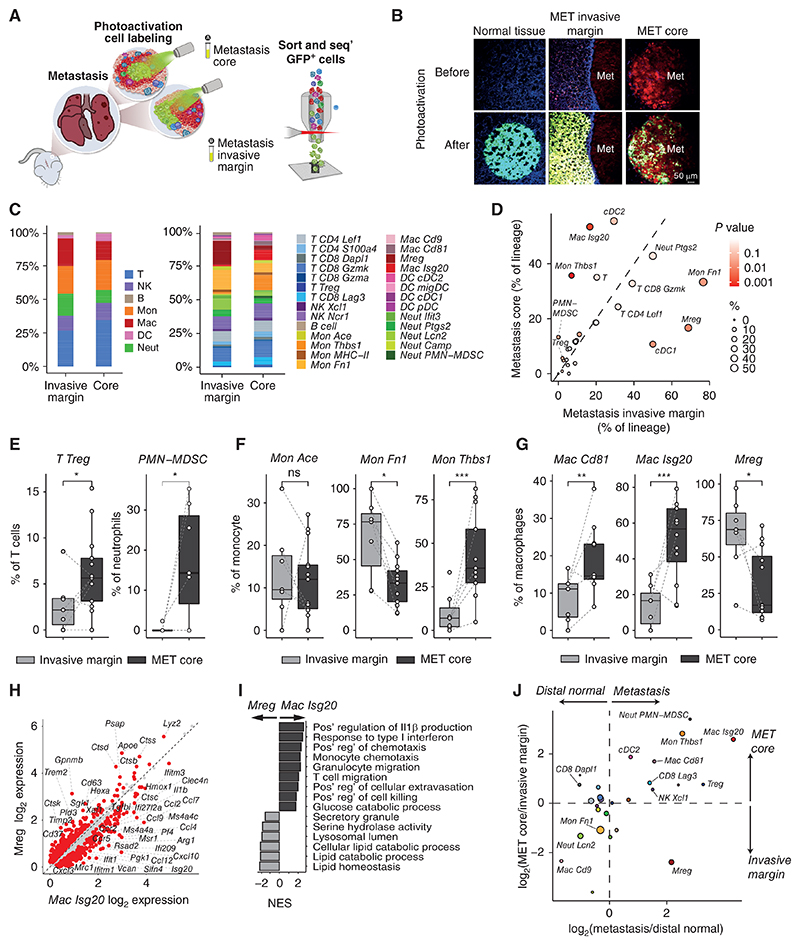

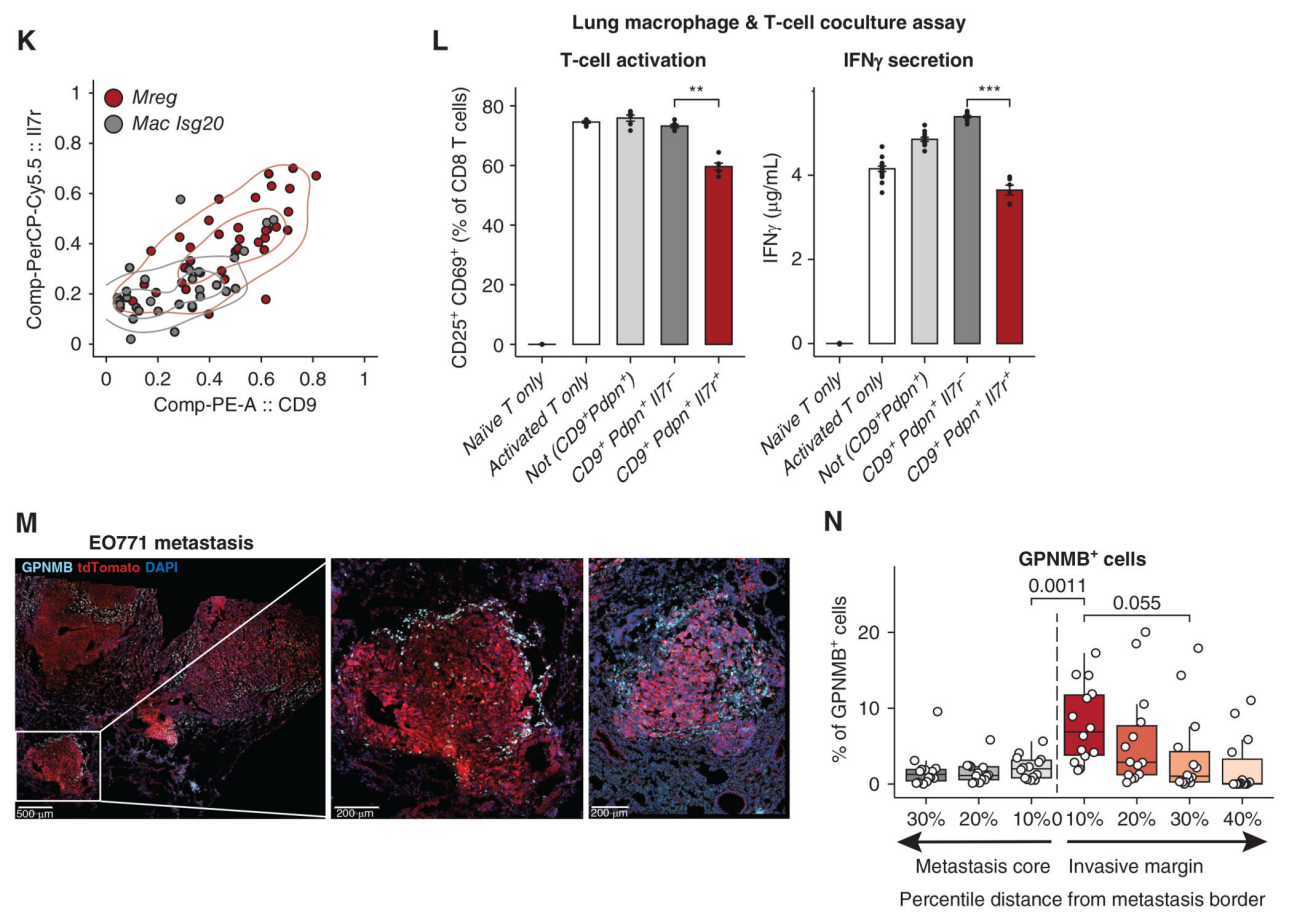
The metastatic invasive margin is populated by suppressive TREM2 macrophages. **A**, Scheme illustrating labeling of cells in metastasis invasive margins and metastatic cores, using PA-GFP. **B**, Representative fluorescent imaging of lung tissue samples pre- and postphotoactivation of a random region in a control sample, and the metastasis invasive margin or core in metastasis-bearing mice. **C**, The cumulative fraction of the different main immune cell lineages (left) and subtypes (right). **D**, Fractions of immune cell lineages (from total) or subtypes (from their respective lineage), averaged over invasive margin (*x*-axis) or metastasis core (*y*-axis) samples. Size indicates the average of x and y. Color gradient depicts *P* value of the two-sided *t* test between x and y, accounting for sample variation. **E**, Fractions of *Tregs* and *PMN-MDSCs* from their respective lineages. **F**, Fractions of the indicated monocyte subtypes from total monocytes. **G**, Fractions of the indicated macrophage subtypes from total macrophages. **H**, Comparison of macrophage subtypes gene expression (log_2_ normalized). Differentially expressed genes (DEG) are colored in red and leading DEGs are labeled. **I**, Enriched gene ontology terms in macrophage subtypes. Normalized gene ontology term enrichment score (NES) is shown on the *x*-axis. For all terms, *P*_adj_ < 0.05. **J**, Cell subtype enrichment in metastasis over distal normal tissues (*x*-axis), compared with metastasis core over invasive margin (*y*-axis). **K**, CD9 and IL7R flow cytometry protein expression values (index sorting) of cells that were annotated as *Mreg* or *Mac Isg20* by following scRNA-seq. **L**, Activated T cells (or not activated control) were cocultured with the lung-derived macrophage populations (CD45^+^ CD11b^+^ Ly6g^−^, and the indicated gate). Quantification of activated CD8 T cells (CD25^+^ CD69^+^, left), and IFNγ secreted in supernatant (right). **M**, Representative immuno-fluorescence imaging of EO771 breast cancer lung metastasis, stained for GPNMB (Cyan). Tumor cells shown in red (tdTomato), nuclei shown in blue (DAPI). **N**, Quantification of the percentage of GPNMB^+^ cells at the metastasis core and invasive margin, at different distances from metastases’ boundary. Two-tailed paired *t* test was used. In boxplots, the center line represents the median, the box limits denote the 25th to the 75th percentiles, and the whiskers represent the minimum and maximum values.

An association between the spatial location and the activity of immune cells within the tumor microenvironment has been demonstrated across multiple cancer types and models (27, 43–45). In several cancer types and murine models, the tumor-invasive margin holds specific cell populations distinct from the tumor core (28, 46). We therefore next asked whether similar differences are operative within metastatic lesions. Our model of spontaneous metastasis enabled us to mark cells located not only in the core of the metastases but also in the invasive margin—the area surrounding the tdTomato-labeled metastases (Fig. 5A and B).

We found the core and invasive margins of metastases to be distinct niches, displaying different immune compositions (Fig. 5C and D; Supplementary Fig. S6A). Within the T-cell compartment, which was overall diminished in metastasis tissues (Supplementary Fig. S4B), *Tregs* were enriched in the core compared with the margin. Interestingly, this was not the case for dysfunctional *CD8 Lag3* or other T-cell populations, as well as the overall T-cell lineage (Supplementary Fig. S6B). *Tregs* and *PMN-MDSCs*, which were enriched in metastatic tissues compared with distal normal, were in fact nearly exclusive to the metastatic core compared with the invasive margin, suggesting unique signaling in these distinct metastatic niches (Fig. 5E).

Within monocytes, the *Mon Thbs1* population was highly concentrated in the metastasis core, whereas *Mon Fn1* was dominant in the invasive margin of metastatic lesions (Fig. 5F). A similar switch occurred between cDC2 cells, which were enriched in the core compared with cDC1 in the invasive margin (Supplementary Fig. S6C). Importantly, when we analyzed the differences in gene expression between both DCs and monocytes in the metastatic core compared with marginal area, we found that cells from metastasis core exhibited elevated IFN-I signature genes, such as *Isg20, Ifitm3*, and *Ly6a* (Supplementary Fig. S6D–S6H). Interestingly, although an IFN-I stimulated DC population has recently been shown to stimulate CD8^+^ T cells and support antitumor immunity (33, 47), other studies suggested that persistent IFN-I signaling in cancer cells contribute to resistance to antitumor immunity (48, 49).

Macrophage subpopulations also presented a divergence between the metastatic core and margin, with the *Mac Isg20* population dominating the core, while *Mregs* dominating the margin (Fig. 5G). *Mac Isg20* are characterized by the expression of neutrophil chemoattractants *Cxcl1/Cxcl2/Cxcl3*, previously associated with metastasis formation (50), C-Type lectin domain family members *Clec4d* and *Clec4n, Il1b*, and IFN-I signaling genes. Our analysis revealed that *Mreg* macrophages expressed potent suppressive immune checkpoints including *Trem2, Gpnmb*, and *Cd63* (refs. 32, 51; Fig. 5H). Compared with the glucose catabolism-oriented *Mac Isg20, Mregs* are biased to lipid catabolism, and expressed cathepsins B, D, K, and S at elevated levels as previously reported in TAMs (ref. 52; Fig. 5I). Macrophages from metastasis core demonstrated enrichment of chemokine signaling, chemotaxis, Il1β production, and IFN-I response compared with invasive margin macrophages (Supplementary Fig. S6I and S6J). Further supporting the importance of these subpopulations, an analysis of cell–cell communication, comparing the core and the invasive margin revealed that the most prominent difference in the interaction network was due to macrophage and monocyte signaling (Supplementary Fig. S6K and S6L). Specifically, monocytes and macrophages in the metastasis core had increased expression of *Ccl2, Ccl7*, and *Ccl12*, which coincided with an increase in *Ccr2* expression. The *Ccl6/9*–*Ccr1* signaling axis between monocytes, macrophages, and neutrophils was also upregulated in the metastasis core. This axis can also act directly on cancer cells expressing the *Ccr1* receptor, promoting their retention in the lungs (53).

To better elucidate the differences in the composition of the metastatic core and invasive margin, we performed a cellular module analysis, in which we correlated the frequency of cell populations across cells from the metastasis core and invasive margins, revealing four cell modules. Module 1 consisted of the invasive margin–enriched *cDC1, Mon Fn1, Mac Cd9*, and *Mreg*. Module 2 consisted of the core-enriched *Treg, cDC2, Mon Thbs1*, and *Mac Isg20*. IFN-I signaling has a range of effects on the different cells in the TME (54), and can either increase antitumor immunity (55) or in case signaling is persistent result in a state of immune dysfunction (56). Notably, the core-enriched cell modules all had in common the upregulation of IFN-I signaling genes, indicating this as a major feature in the metastasis core (Supplementary Fig. S6M).

In order to identify cell populations that are spatially distinct in lung metastatic niches, we performed an analysis that compared the enrichment in metastatic versus distal normal tissues, to the enrichment in the core versus the invasive margin. Interestingly, the dynamics of the cell populations were correlated, with the exception of *Mregs*. In other words, all of the metastasis-enriched populations were also enriched in the metastasis core, except for *Mregs*. This finding implies that the *Mreg* macrophage population, surrounding metastases rather than at their core, is a hallmark of metastasis-bearing lung tissues (Fig. 5J). Interestingly, the *Mreg* population was also recently observed to be highly predictive for poor response to immunotherapy (57). Next, we investigated whether the specific spatial domains of the macrophage populations in the metastatic lung tissues are related to distinct functions in their modulation of T-cell activation. We therefore combined flow cytometry analyses of protein expression with scRNA-seq using index sorting to define an effective panel for *Mreg* isolation and functional analysis from metastatic lung tissue.

We found that *Mregs* isolated from metastatic lung tissues displayed coexpression of specific markers, including CD9, PDPN, TREM2, GPNMB, SPP1, and IL7R. In comparison, the *Mac Isg20* cells did not exhibit this coexpression pattern (Fig. 5K; Supplementary Fig. S7A). Importantly, this population was also specific to metastatic tissues (Supplementary Fig. S7B). We sorted myeloid cells (CD11b^+^ Ly6G^−^) from EO771 spontaneous lung metastases, isolating double-positive cells for CD9^+^PDPN^+^ to IL7R high (*Mreg*), IL7R low (Supplementary Fig. S7C), and a third control population of cells that were not (CD9^+^PDPN^+^) double positive. To assess the functional role of *Mreg* cells, these three populations were cocultured with activated T cells for 72 hours, and T-cell activation and IFNγ secretion were analyzed (Supplementary Fig. S7D). The results indicated that only the *Mreg* macrophage population was capable of suppressing T-cell activation and the secretion of IFNγ (Fig. 5L). This finding highlights the critical role of *Mregs* as the dominant macrophage population in the invasive margins of murine lung metastases and underscores their ability to dampen immune activation at the stromal–tumor front.

To further validate the spatial distribution of *Mreg* macrophages in metastases, we performed immunofluorescence imaging of EO771 breast cancer lung metastases, using GPNMB as a marker for the *Mreg* population (Fig. 5M; Supplementary Fig. S7E). Indeed, GPNMB^+^ cells were predominantly detected at the metastases invasive margins. Further validating our findings, quantification of the percentage of GPNMB^+^ cells at defined distance intervals from the metastasis border confirmed that *Mregs* were highly enriched in the metastasis invasive margin (Fig. 5N; Supplementary Fig. S7F). In addition, we observed the same phenotype using TREM2 as a marker for imaging *Mreg* of either EO771 or 4T1 breast cancer lung metastases (Supplementary Fig. S7G), confirming the unique spatial distribution of *Mregs*.

These results are in line with our previous studies showing TREM2^+^ macrophages in fat tissue or disease-associated microglia residing in the surroundings of pathologic tissues, cloaking them from further immune insult (58, 59). CD9^+^TREM2^+^ macrophages expressing GPNMB, SPP1, FABP5, and CD63 were reported in murine and human pulmonary fibrosis, enriched at the edges of scars (60). Importantly, GPNMB^+^ TAMs were found to be located closer to the margin of colorectal liver metastasis (61), and we found that murine lung metastasis *Mregs* share the same unique gene-expression signature with this population (Supplementary Fig. S7H).

In summary, these results underline the myeloid compartment as the major feature distinguishing between the immune milieu at the metastatic core and invasive fronts, suggesting a role in immune suppression and ECM remodeling at the invasive margin of metastases, thus cloaking the metastatic lesions from antitumor immunity and promoting metastasis expansion.

**Figure 6:**
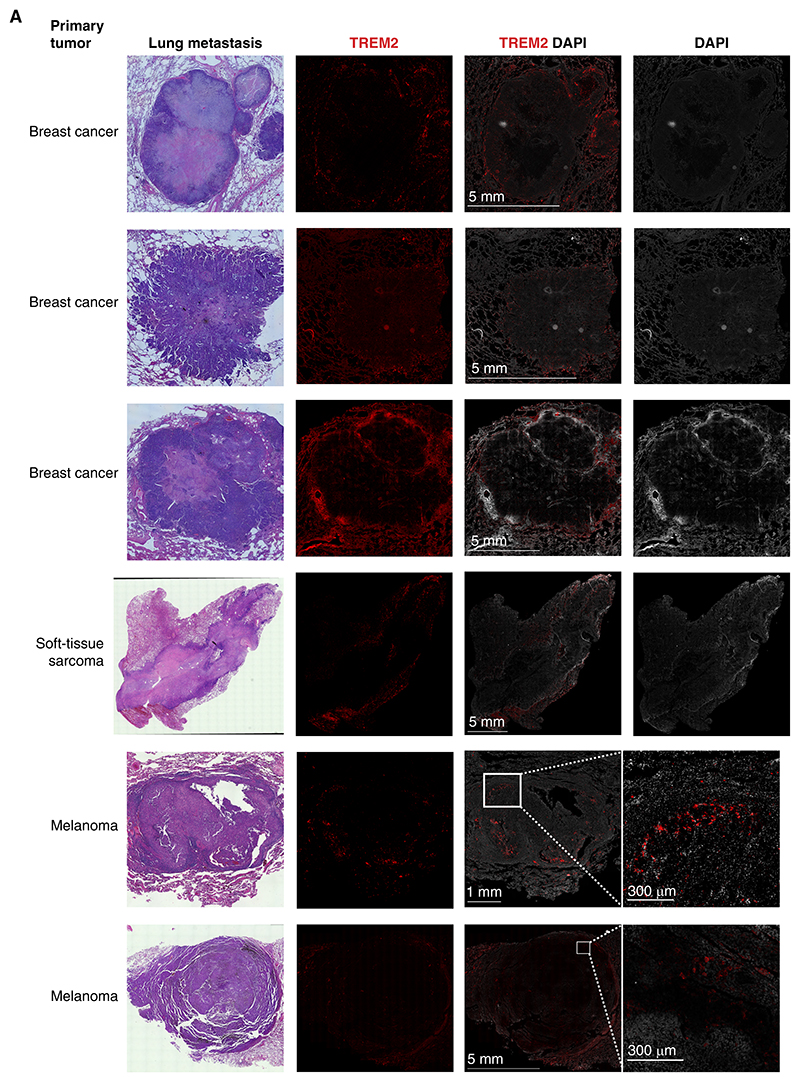
*Mregs* accumulate at the invasive margin of human lung metastases. **A**, H&E and immunofluorescent images of patient lung metastasis sections. Each patient’s primary tumor diagnosis is indicated on the left. FFPE sections were stained using DAPI and rabbit anti-TREM2 (clone D8I4C, Cell Signaling Technology; #91068). Image acquisition was performed using a Leica DMi8 widefield microscope with a 20× objective (Leica Microsystems).

Finally, we asked whether our findings that TREM2^+^ *Mregs* were enriched at the invasive margin of breast cancer lung metastasis models are also operative in human lung metastasis. To this end, we performed immunofluorescence imaging of lung tissue sections from patients with lung metastasis from multiple cancer types, including breast, melanoma, and soft-tissue sarcoma. Analysis of *Mreg* staining indicated that, similar to our findings in murine lung metastasis, TREM2^+^ cells accumulated at the invasive margin of human lung metastases (Fig. 6A), suggesting that niche-specific spatial distribution of macrophage subpopulations plays a key role also in the shaping of human immune metastatic niches.

## Discussion

In this study, we characterized the temporal and spatial changes in the immune microenvironment of breast cancer lung metastases during spontaneous metastatic relapse in immune-competent mice. We identified marked differences between the microenvironments of the primary tumor and metastases. Moreover, we charted the dynamic changes in the immune landscape during the formation of the premetastatic niche and in the early stages of metastatic progression, while highlighting the unique immune functional subpopulations at distinct micro-niches in metastatic lungs.

Immunotherapies have revolutionized the treatments of many cancer types (62). However, breast cancer is generally regarded to be a “cold” tumor, and most immunotherapy strategies have proven ineffective, with one exception (63). Therefore, a better understanding of the immune landscape in the TME of breast tumors is crucial for improving therapeutic strategies. In recent years, multiple studies have thoroughly characterized the immune and stromal microenvironment in breast cancer (27, 64–66), but these studies have focused on the primary tumor site. Because breast tumors are typically resected, and patients are treated with targeted therapeutics for metastatic disease, it is important to understand the metastatic microenvironment, which we show to be distinct from that of the primary tumor.

The mTME in the lungs demonstrated increased infiltration of both activated and dysfunctional CD8 T cells, and diminished levels of Tregs. The myeloid compartment in the mTME was also discrete, with an altered phenotype of monocytes and macrophages, unlike the MHC-II expressing TAMs found in the primary tumor. Our findings indicate that preclinical testing of immunotherapies targeting either lymphocytes or myeloid cells should take place in the appropriate organ-specific tumor niche, as the landscapes of the target cells can vary greatly between tumor sites. Considering each organ has a unique TME, it would be beneficial for precision therapeutics to perform further profiling of additional metastatic organs of breast cancer, including bone, liver, and brain.

It is well accepted that many of the changes that enable metastatic progression are instigated systemically and precede the formation of clinically relevant metastases (12). Utilizing clinically relevant models of spontaneous metastasis following resection of the primary tumor enabled us to characterize the alterations in the immune landscape of the lung at the premetastatic stage. We found that recruitment of nonclassic, inflammatory monocyte, and neutrophil subpopulations are the main immune features of this stage. These findings are in agreement with previous studies showing metastasis-associated expansion of neutrophils and monocytes (18, 35, 67), as well as their ability to promote metastasis via suppression of cytotoxic T cells. Interestingly, expansion of these myeloid cells was reported in other cancer types, implicating it as a general lung metastasis mechanism, rather than a breast cancer–specific process. The expanded monocyte and neutrophil subpopulations expressed programs of ECM remodeling and NET formation and communicated via the *Ccl6–Ccr1/2* signaling axis. Functionally, we found that the noncellular fraction containing secreted factors from Pre-MET lungs induced migration of bone marrow–derived monocytes and granulocytes, which was at least partially mediated by *Ccl6–Ccr1/2* signaling, as inhibition of this pathway decreased chemoattraction of these BM-derived cells. Thus, our findings suggest that manipulating the signaling pathways that direct the formation of a hospitable metastatic niche may prove to be an efficient strategy to prevent metastatic relapse.

Comparison of distal, seemingly normal lung tissue from metastases-bearing animals with normal lungs or to isolated metastatic lesions revealed that distal “normal” tissues were more similar to the metastatic tissue than to normal lungs. Thus, although metastatic progression entails the formation of defined tumor lesions in the lungs, the entire organ is diseased. Nevertheless, we found a specific immune cell module that was exclusive to metastatic lesions, consisting of cells of a suppressive nature. Monocytes and macrophages in metastatic tissues were the most altered, skewed toward programs of glycolysis, angiogenesis, and IFN-1 signaling. Furthermore, *ex vivo* assays demonstrated that metastatic lung tissues were better capable of attracting monocytes, and their macrophages inhibited T-cell proliferation, indicating their suppressive nature.

Using photoactivable-GFP mice as recipients for tumors enabled us to further dissect the spatial composition of mTME. Isolation and profiling of the metastases core revealed which of the cells enriched in metastasis lesions (compared with distal normal tissues) were concentrated in the tumor core (e.g., *Treg, PMN-MDSCs, Mon Thbs1, Mac Isg20*). In contrast, we found that the invasive margins of metastatic lesions were highly infiltrated by *Trem2*^+^ *Mreg. Mreg* (or TREM2^+^ macrophages) were reported as enriched in tumors in numerous murine models and clinical indications including breast, lung, and colon (68, 69). The location of inhibitory macrophage subsets at primary breast tumor invasive margins has been previously demonstrated PT, but not in metastasis. CD68^+^ and CD163^+^ macrophages were found to be enriched in the invasive front of luminal ductal carcinomas (70). A recent study of primary breast cancer tumors found that FOLR2^+^ tissue-resident macrophages are present in the tumor stroma, as opposed to TREM2^+^ macrophages that concentrate in tumor nests and margins (28). Similar to these observations, we found that TREM2^+^ *Mregs* were most prominent in the invasive margin of early lung metastases, unlike all other metastasis-enriched subpopulations that were located in the metastases core. Moreover, functional assays indicated that this *Mregs* subpopulation, unlike other macrophage populations, suppresses T-cell activation and IFNγ secretion. This finding, as well as the increased ECM remodeling pathway upregulated in *Mregs*, could imply that this macrophage population supports tumor growth and progression via cloaking of the metastatic site and modification of the tumor microenvironment at the invasive margin. Indeed this would be supportive of the recent finding that the *Mreg* population was observed to be the most predictive cell type for defining patients with poor outcome and response to immunotherapy (57). Importantly, our findings in the murine breast cancer models were confirmed in patient samples. Imaging of lung metastasis from patients with cancer revealed that TREM2^+^ cells reside at the invasive margin of metastases in both breast cancer and other primary tumor sources such as melanoma and soft-tissue sarcoma. Our findings are in line with previous observations of GPNMB^+^ cells enriched in the invasive margin of liver metastases, and other pathologic conditions such as scar formation (60, 61). Together, these results indicate that the spatial distribution of *Mregs* may be a conserved pathologic mechanism of a failed immune response. Future studies could test the correlation of the spatial distribution of specific macrophage populations with disease progression and may serve as a clinical feature to be monitored in clinical trials targeting TAMs.

In summary, we characterized the metastatic microenvironment in breast cancer lung metastasis, achieving a single-cell resolution analysis with spatial segmentation. We identified unique alterations in the immune composition over both temporal and spatial dimensions in the metastatic niche. Overall, the results of this study provide a deeper understanding of the immune microenvironment in metastatic lungs and highlight the importance of macrophages in promoting or inhibiting tumor growth and progression. These findings may have implications for the development of novel therapeutic strategies that target specific macrophage populations in the metastatic niche. Our findings highlight the importance of gaining better knowledge of organ-specific immune changes to enable the development of more accurate and efficient immunotherapies to inhibit metastatic relapse.

## Methods

### Animals

All animal procedures included in the study were granted ethical approval by the Tel Aviv University Institutional Animal Care and Use Committee. All animals were maintained within the Tel Aviv University–specific pathogen-free facility. Mice were provided with food and water ad libitum and housed under a strict 12-hour light–dark cycle. BALB/c and C57BL/6 mice were purchased from Harlan. PA-GFP mice (71) were purchased from the Jackson Laboratories. Mice used for experiments were females at 8–10 weeks of age, unless otherwise stated.

### Cell Lines

The EO771-tdTomato and 4T1-tdTomato cell lines were generated by infection of the original EO771 (ATCC; cat. # CRL-3461, RRID:CVCL_GR23) or 4T1 (ATCC; cat. # CRL-2539, RRID:CVCL_0125), a mouse breast cancer cell line, with a tdTomato2 lentiviral vector, and cultured in Hygromycin selective media. MC38 murine colon adenocarcinoma cell line culture and tumor growth and scRNA-seq were performed as described previously (72). Cells were cultured in 100-mm tissue culture plates in an incubator with humidified air and 5% CO_2_ at 37°C. Cell lines were validated for lack of *Mycoplasma* infection using primers for *Mycoplasma*-specific 16S rRNA gene region (EZPCR Mycoplasma Kit; Biological Industries).

### Orthotopic Tumor Transplantations

Tumor cells (5 × 10^5^ EO771-tdTomato cells or 2 × 10^5^ 4T1-tdTomato cells) were suspended in PBS and mixed 1:1 with Matrigel (BD Biosciences, 354230). A total of 100 μL of cell mixture was injected into the right inguinal mammary glands of 8-week-old female PA-GFP C57BL/6 or BALB/c mice. Tumors were resected 3 weeks following the injection, and CT imaging of lungs was performed once a week until metastases appeared. Mice were anesthetized and lung perfusion was performed.

### Ex Vivo *Photoactivation and Image Acquisition of Lung Niches*

Photoactivation and imaging were performed as described previously (26). Following perfusion and dissection of the intact lungs, lung lobes were separated (at this stage metastatic sites were often seen by the eye as distinct red dots). Tissues were viewed in the microscope to identify metastases or validate the lack of tdTomato signal in distal normal, relapse-free, or Pre-MET tissues. Note that as it is widely known that cancer cells can also lose fluorescent marker expression over time, we could not rule out that no tumor cells are found in any tissue. Therefore, our classification of tissues asks whether there are macrometastases that are clearly defined or the presence of a primary tumor (premetastatic niche). Because we attempted to characterize metastasis as early as possible after their first detection, in some cases, only one metastatic lesion was found and therefore we were able to only label either the core or the invasive margin (Supplementary Table S2).

### Tissue Processing to Single Cells for scRNA-seq

Lung tissues or primary EO771 tumors underwent mechanical and enzymatic digestion for 15 minutes at 37°C (gentleMACS C tube, Miltenyi Biotec Inc.; 0.1 mg/mL DNase type I (Roche), and 1 mg/mL Collagenase IV (Worthington) in RPMI-1640). Lungs were dissociated with the m_lung program, and tumors with the h_tumor program. Cells were then filtered through a 100-μm cell strainer, washed with cold PBS, and centrifuged (5 minutes, 4°C, 300× *g*).

### Single-Cell Sorting

Following tissue digestion, cells were washed and resuspended in cold FACS buffer (0.5% BSA and 2 mmol/L EDTA in PBS), and incubated with fluorophore-conjugated antibodies (CD31-PE/Cy7, BioLegend 102417, RRID:AB_830756; CD45-APC/Cy7, BioLegend 103115, RRID:AB_312980). Following the staining cells were filtered through a 70-μm strainer. Before sorting, cells were stained with DAPI to exclude dead/dying cells. Cell sorting was performed using a BD FACSAria Fusion flow cytometer (BD Biosciences), gating for CD31^−^CD45^+^ cells, or when isolating photoactivated cells for CD31^−^GFP^+^ (after exclusion of dead cells and doublets).

### SPID-seq

SPID-seq is a method for plate-based scRNA-seq, calibrated for increased efficiency and high throughput, enabling the rapid profiling of many samples and sorting gates/cells per sample (Supplementary Fig. S8A and S8B). Our analyses verified that SPID-seq enabled the identification and distinction of cell types and subpopulations (Supplementary Fig. S8C and S8D). Single cells were sorted into 384-well capture plates (Eppendorf twin.tec PCR Plates 384 TT38440SC-EP) containing 20 nmol/L barcoded poly(T) reverse-transcription (RT) primers for scRNA-seq in 100 nL of lysis solution composed of 0.5 U/μL Ribolock (Thermo Fisher Scientific EO0382) and 0.2% Triton X-100 (Sigma-Aldrich, T8787), and 3 μL mineral oil (Sigma-Aldrich, M8410). cDNA is generated and amplified per well by reverse transcription that adds a unique molecular identifier (UMI) and a well barcode (ACTCACTATAGGGGCGAC GTGTGCTCTTCCGATCTxxxxxxxNNNNNNNN TTTTTTTTTTTT TTTTTTTTVN; N-random base, x-Well barcode; Oligo synthesized by Integrated DNA Technologies). A template-switch oligo is incorporated at the cDNA 3′ end (/5-MeIsodC//iso-G//iso-C/AAGCAG TGGTATCAACGCAGAGTACrGrG+G), and used for amplification in a PCR reaction (9 cycles). After dispensing 300 nL of reaction mix the final 400 nL reaction contained: 0.6M Betaine (Sigma-Aldrich B0300), 1.83 mmol/L DTT, 5.78 mmol/L dNTPs (Sigma-Aldrich, NU-1005XS), 0.8 μmol/L template-switch oligo), 6.65M MgCl2, oligos CATCGATGAATTCTCTGTCGgcaagtggAAGCAGTGGTATC AACGCAGAGT, and /5Biosg/ACTCACTATAGGGGCGACGTGT at 0.2 μmol/L each, 0.001% w/v Bromophenol Blue, 0.02 U/μL Terra PCR Direct Polymerase Mix and 0.14× Terra PCR buffer (Takara Bio, 639271), 1 U/μL SuperScript II Reverse Transcriptase and 0.64× first-strand RT buffer (Thermo Fisher Scientific, 18064014), nuclease-free water (Sigma-Aldrich, W4502-1L). The content of each plate was pooled and purified using AMPure XP SPRI beads (Beckman Coulter, A63881) at a 0.7× ratio, then fragmented using homemade Tn5, adding an Illumina nextera read 1 sequence. Fragmented DNA was purified (0.7×), and a plate-indexing PCR reaction was performed using KAPA HS mix (Roche, KK2601), with 0.3 μmol/L of each index oligo: i7-CAAGCAGAAGACGGCATACGAGATxxxxxxxxGTG ACTGGAGTTCAGACGTGTGCTCTTCCGATCT and i5-AATGATA CGGCGACCACCGAGATCTACACxxxxxxxxTCTTTCCCTACACGA CGCTCTTCCGATCT. DNA was purified and double-size selected using SPRI beads at 0.6× and then 0.85× ratios. Each plate library was tested for quality and DNA concentration. Sequencing libraries were pooled at equimolar concentrations and sequenced using an Illumina NextSeq 500 or NovaSeq 6000 sequencer, at a sequencing depth of 10K–50K reads per cell. Reads are condensed into original molecules by counting the same UMIs. We used statistics on empty-well spurious UMI detection to ensure that the batches we used for analysis showed a low level of cross single-cell contamination (less than 3%). Reads were processed as previously described (73). Reads were mapped to murine reference genome mm10 using HISAT (version 0.1.6); reads with multiple mapping positions were excluded. Reads were associated with genes if they were mapped to an exon, using the UCSC genome browser for reference. Exons of different genes that shared genomic positions on the same strand were considered a single gene with a concatenated gene symbol.

### Migration Assay

Bone marrow–derived cells (5 × 10^5^) were placed at the upper chamber of 24-transwell membrane plates with 5-mm pores (Corning; CLS3421). Lung homogenate supernatant from control mice lungs, premetastatic lungs, whole metastases-bearing lungs, or dissected distal normal areas and metastatic lesion areas from metastases-bearing lungs, were placed at the bottom chamber. In appropriate wells, anti-CCL6 antibody (R&D Systems, MAB487, RRID:AB_2071563) was added to the bottom chamber. Migrated cells were stained for CD11b^+^Ly6C^int^Ly6G^+^ (Granulocytes) or CD11b^+^Ly6C^+^Ly6G^−^ (Monocytes) and counted by flow cytometry (Cytoflex LX, Beckman Coulter).

### Macrophage–T-cell Coculture Activation Assay

Macrophages from the different lung lesions were sterile sorted into C10 medium using Cd45^+^ F4/80^+^ gating. For specific macrophage populations isolation, metastatic lung tissues from 5 different mice were inspected by flow cytometry, and combined for the sorting of live (sytox negative), CD45^+^, CD11b^+^, Ly6g^−^, and either CD9^+^ PDPN^+^ IL7r^+^, CD9^+^ PDPN^+^ IL7r^−^, or not (CD9^+^ PDPN^+^). T cells were isolated using a Pan T-cell isolation kit (Miltenyi Biotec, 130-095-130) from a spleen of a 11-week-old WT female (C57BL/6) mouse according to the kit guidelines. T cells were then labeled with cell proliferation dye eFluorTM 450 (Thermo Fisher, 65-0842-85) according to the manufacturer’s guidelines. For activating the T cells in the coculture wells, a 96-well tissue culture treated plate was precoated with 1 μg Ultra-LEAF purified anti-mouse CD3 per well (BioLegend, 100340, RRID:AB_11149115) for two hours at 37°C and then washed twice with PBS. Cells were then cocultured in a 1:3 ratio (macrophage:T-cell) in 100 μL C10 medium per well, supplied with 2 μg/mL Ultra-LEAF purified anti-mouse CD28 (BioLegend, 102116, RRID:AB_11147170) and kept in a 37°C, 5% CO_2_ incubator. Cells were harvested after 72 hours, and cell proliferation was analyzed using an LSRII FACS analyzer (BD). Alternatively, after harvesting, cells were stained with anti-CD25 (clone PC61, BioLegend 102007, RRID:AB_312856) and anti-CD69 (clone H1.2F3, BioLegend 104514, RRID:AB_492843) for assessment of cell activation (Fig. 5L). Mouse interferon-gamma secretion was measured in the coculture media by an ELISA kit (BioLegend, 430801, RRID:AB_2893366).

### Immunostaining

#### Mouse Tissue Sections

Lungs were harvested, washed in PBS, and incubated for 4 hours in 4% PFA (Electron Microscopy Sciences). Lungs were transferred to 30% sucrose for 48 hours and then embedded in Optimal Cutting Temperature compound (Tissue-Tek) on dry ice and stored at −80°C. Serial sections were obtained to ensure equal sampling of the examined specimens (10 mm trimming).

#### Mouse Tissue Immunofluorescence

Frozen lung tissue sections were incubated at 60°C for 30 minutes, washed with PBST, and incubated with 3% H_2_O_2_ for 12 minutes to block endogenous peroxidase activity. Slides were washed twice with DDW for 5 minutes, incubated with 0.5% triton X-100 (Sigma-Aldrich, T8787) for 10 minutes and washed 3 times in TBS-T, followed by one wash with PBS. Then, slides were blocked with CAS-Block (Thermo Fisher, 008120) for 10 minutes and incubated overnight at 4°C with the following primary antibodies: rabbit anti-mouse GPNMB (Abcam, ab234529, RRID: AB_3064846), rat anti-mouse F4/80 (Bio-Rad, MCA497R, RRID:AB_323279), sheep anti-mouse TREM2 (R&D Systems, AF1729, RRID:AB_354956), and goat anti-mouse tdTomato (LSBio, LS-C340696, RRID:AB_2819022), diluted in antibody diluent (Abcam, ab64211) with 0.02% triton. Slides were washed and incubated for 1 hour at room temperature with the following secondary antibodies diluted 1:200: Peroxidase-AffiniPure Donkey Anti-Rabbit (Jackson ImmunoResearch, RRID:AB_10015282), Peroxidase-AffiniPure Donkey Anti-Sheep (Jackson ImmunoResearch, 711-035-147, AB_2340710), Alexa Fluor 488 goat anti-rat (Thermo Fisher, A11006, AB_2534074) and Rhodamine (TRITC)-AffiniPure Donkey Anti-Goat (Jackson ImmunoResearch, 705-025-003, RRID:AB_2340388). Slides were washed, and the slides that were incubated with the peroxidase antibodies were incubated with opal 650 reagents (AKOYA, FP1496001KT) diluted 1:400 in amplification diluent (AKOYA, FP1135). Slides were washed again and incubated with DAPI (Sigma-Aldrich, MBD0015), mounted with Fluoroshield (Sigma-Aldrich, F6182), left to dry for 2 hours at room temperature and stored at 4°C.

Images were acquired using Leica TCS SP8 Confocal Microscope Model Number S/N 8100000117 or with an Aperio Versa 200 slide scanner. Brightness and contrast were adjusted equally in all images. Quantitative analyses were performed using IMARIS 9.5 imaging software.

#### Human Sections

Patient FFPE sections were obtained from the Tel Aviv Sourasky Medical Center Institutional BioBank, and the study was approved by the Institutional Review Board of (TLV 0417-20) and the ethical commission of Hadassah Medical Center (HMO 0235-21), and conducted in accordance with the Declaration of Helsinki protocol. All patients provided their written informed consent.

#### Human Tissue Immunofluorescence

. For spatial examination in human specimens, FFPE sections were baked at 37°C overnight, deparaffinized in xylene, and rehydrated in decreasing concentrations of ethanol. Tissue sections were incubated in citrate buffer (pH 6) for antigen retrieval at 95°C for 30 minutes. After three PBS washes, we added blocking buffer (5% donkey serum in PBST and 0.1% Triton X-100) for 1 hour at room temperature. After blocking, primary antibodies were incubated at 4°C overnight: rabbit anti-TREM2 (clone D8I4C, 1:200 dilution, Cell Signaling Technology; #91068, RRID:AB_2721119). After three PBST washes (0.01% Tween-20; Sigma-Aldrich), corresponding secondary antibodies were used for 1 hour at room temperature. After three PBST washes, a TrueVIEW autofluorescence quenching kit (Vector Laboratories; SP-8400) was applied before nuclei staining with DAPI for 6 minutes and coverslips were then mounted on slides with anti-fade mounting medium in the autofluorescence quenching kit, and mounted slides were kept in the dark. Image acquisition was performed using a Leica DMi8 widefield microscope with a 20× objective (Leica Microsystems).

### Bioinformatic Analysis

#### Mapping

For low-level processing and filtering, sequences were mapped to the mouse genome (mm10), demultiplexed, and filtered, extracting a set of UMIs that define distinct transcripts in single cells for further processing. Mapping of reads was done using HISAT v.0.1.6; reads with multiple mapping positions were excluded. Reads were associated with genes if they were mapped to an exon, using the UCSC genome browser for reference.

#### Quality Control

Cells with fewer than 500 UMIs were discarded from the analysis. All downstream analyses were performed in R.

#### Unsupervised Clustering Analysis and Annotation

The metacell pipeline was used to derive informative genes and compute cell-to-cell similarity, to compute K-nn graph covers and derive the distribution of RNA in cohesive groups of cells (or metacells), and to derive strongly separated clusters using bootstrap analysis and computation of graph covers on resampled data. Default parameters were used.

Metacells were further clustered using hierarchical clustering with post hoc manual fine-tuning.

To annotate clusters, we implemented a supervised approach using the curated list of marker genes.

#### Dimensionality Reduction

Two-dimensional visualization of the metacell structure was performed as previously described (31). In short, a symmetric graph is constructed over all metacells, by thresholding over the coclustering statistics (indicating how cells from two distinct metacells are likely to be clustered together). This results in a graph with maximum degree, D, and any number of connected components. MetaCell computes coordinates for each metacell by applying a standard force-directed layout algorithm to the graph. It then positions cells by averaging the metacell coordinates of their neighbor cells in the K-nn graph, but filters neighbors that define a metacell pair that is not connected in the graph.

#### Compositional Analysis

The cluster composition of each sample was calculated separately. The enrichment score used for bubble plots was calculated as the ratio of sample average population fractions from each group, and the bubble size depicts the average of the sample average population fractions in the two groups.

To test if changes in composition were statistically significant, we assumed that cell population fraction, like many other biological parameters, was approximately normally distributed in the population. Therefore, a two-tailed Student *t* test was used to compare fractions in different groups. Often, we were interested in the relative fraction of two specific populations. Therefore, we calculated the log_2_ (fold change) per sample. This estimator is a better statistic for using a *t* test because it overcomes the bias introduced by the interdependency of cell population fractions (that sum up to 100%).

#### Sample PCA

The cell-type makeup of each sample was calculated as a vector of relative fractions. The matrix of these vectors was scaled (but not centered) and PCA was applied using pca() function of the pcaMethods R library.

#### Cellular Module Analysis

To define cellular modules across samples, we used a consensus clustering algorithm for determining cluster membership by stability evidence. Specifically, we used the ConsensusClusterPlus() function implemented in the Consensus-ClusterPlus R library available on Bioconductor. The parameters used were reps = 500, pItem = 0.8, clusterAlg = “hc” and distance = “spearman.”

#### Differential Expression Testing and Marker Gene Detection

To find marker genes, we used the FindAllMarkers() function in Seurat v.4.1.0 with a one-sided Wilcoxon rank-sum test on log-transformed normalized counts. DEGs were selected to be fold change >1.5, Bonferroni-adjusted *P* < 0.05, of which the top-scoring genes were presented.

To compare treatments or cell types, we pooled together cells from all samples of each comparison group and used FindMarkers() with a two-sided Wilcoxon rank-sum test on log-transformed normalized counts. We considered genes that were expressed in >0.01 of cells and with >5 cells with >2 UMIs. DEGs were selected to be |fold change| >1.25, Benjamini–Hochberg-adjusted *P* < 0.05, of which the top-scoring genes were presented. See Supplementary Table S3.

#### GSEA

We used GSEA (RRID:SCR_003199) to detect enriched gene sets in different treatment arms. We applied the Fast GSEA (“fgsea”) package, implemented in R, to sorted gene fold changes generated by the FindMarkers() function from Seurat. Gene sets were drawn from mouse C5 v5p2 gene ontology (GO) collection of the Molecular Signature Database. See Supplementary Table S4.

#### Cell–Cell Interactions Analysis

Cell–cell interactions were inferred using CellChat v.1.6.0 (RRID:SCR_021946), following the official workflow, using as input UMI count matrix of the relevant condition and cell types, and the ligand–receptor pairs curated in the lists “Secreted Signaling,” “ECM-Receptor,” and “cell–cell contact.” For our purposes, we excluded MHC-related pairs.

#### Visualization

Plots were generated in R using ggplot2 (RRID: SCR_014601), CellChat, and ComplexHeatmap (RRID:SCR_017270) R libraries.

## Supplementary Material

Fig. s1

Fig. s2

Fig. s3

Fig. s4

Fig. s5

Fig. s6

Fig. s7

Fig. s8

Table s1

Table s2

Table s3

Table s4

## Data Availability

The data generated in this study are publicly available in Gene-Expression Omnibus (GEO GSE231915).

## Significance

Temporal and spatial single-cell analysis of metastasis stages revealed new players in modulating immune surveillance and suppression. Our study highlights distinct populations of TREM2 macrophages as modulators of the microenvironment in metastasis, and as the key immune determinant defining metastatic niches, pointing to myeloid checkpoints to improve therapeutic strategies.
