# Supplementary material for: Spatial and Temporal Mapping of Breast Cancer Lung Metastases Identify TREM2 Macrophages as Regulators of the Metastatic Boundary
Source: Cancer Discov. Author manuscript; Available in PMC 2025 Jul 22. (PMC7617931; doi:10.1158/2159-8290.CD-23-0299)
Supplement: Fig. s1 [file EMS206810-supplement-Fig__s1.pdf]

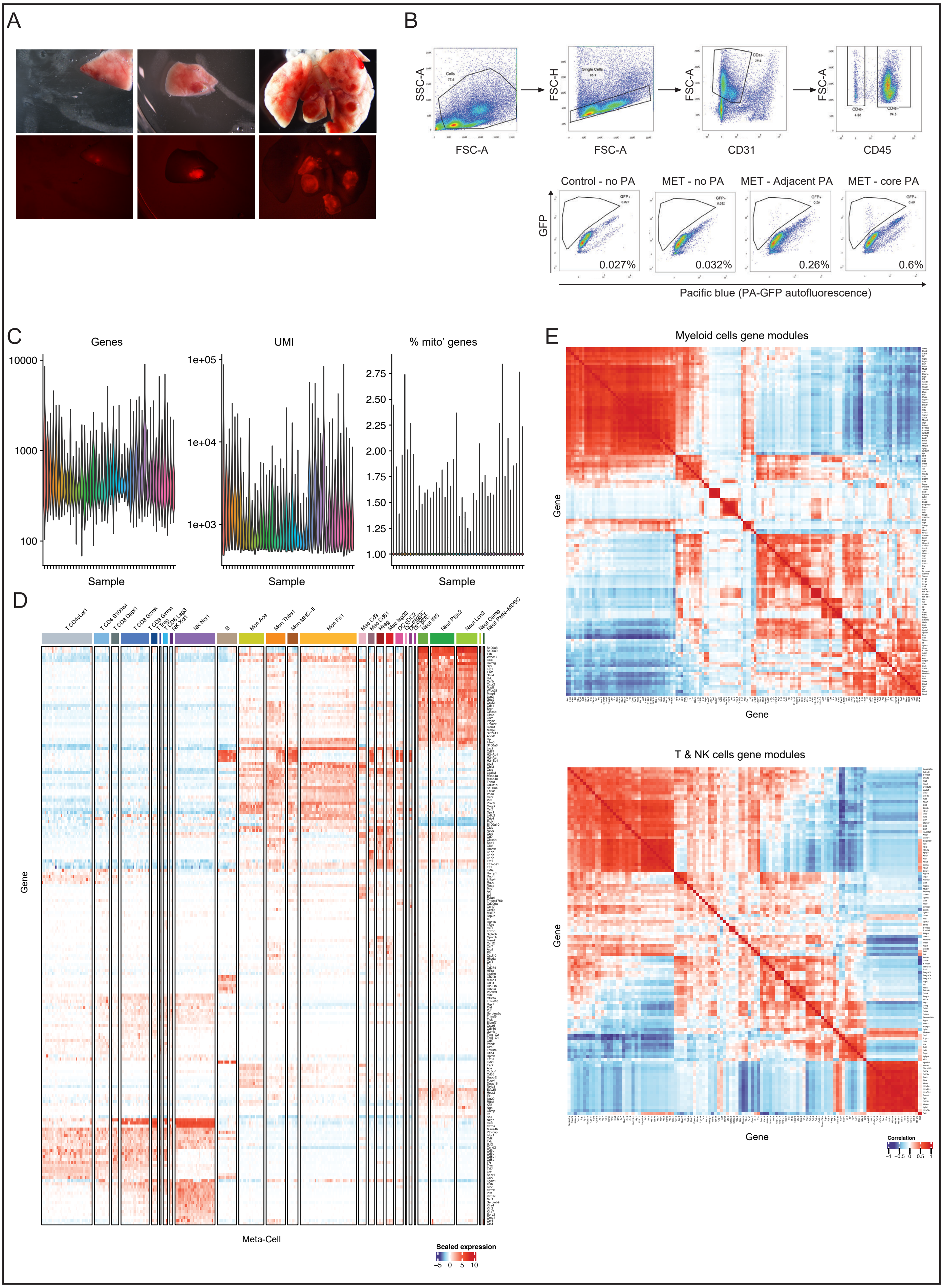

A
B
SSC-A
FSC-A
FSC-H
FSC-A
FSC-A
CD31
FSC-A
CD45
Control - no PA
MET - no PA
MET - Adjacent PA
MET - core PA
GFP
0.027%
0.032%
0.26%
0.6%
Pacific blue (PA-GFP autofluorescence)
C
Genes
UMI
% mito' genes
10000
1000
100
1e+05
1e+04
1e+03
2.75
2.50
2.25
2.00
1.75
1.50
1.25
1.00
Sample
Sample
Sample
D
Gene
Meta-Cell
Scaled expression
E
Myeloid cells gene modules
Gene
Gene
T & NK cells gene modules
Gene
Gene
Correlation

Supplementary Figure 1. Detailed atlas of the immune microenvironment in breast cancer lung metastasis.

A. Bright-field and fluorescent images of lungs with EO771-tdTomato metastatic lesions.

B. Gating strategy used to sort CD45+ immune cells and niche-specific photoactivation labeled cells.

C. The number of genes, unique molecular identifiers (UMI), and percent of mitochondrial gene expression (indicating low-quality cells) per sample.

D. Gene expression profiles of cells grouped by their MetaCell and subpopulation assignment.

E. Gene-gene correlation heatmap of top variable genes within myeloid (top) or T and NK (bottom) MetaCells.
