# Supplementary material for: Spatial and Temporal Mapping of Breast Cancer Lung Metastases Identify TREM2 Macrophages as Regulators of the Metastatic Boundary
Source: Cancer Discov. Author manuscript; Available in PMC 2025 Jul 22. (PMC7617931; doi:10.1158/2159-8290.CD-23-0299)
Supplement: Fig. s2 [file EMS206810-supplement-Fig__s2.pdf]

Supplementary Figure 2

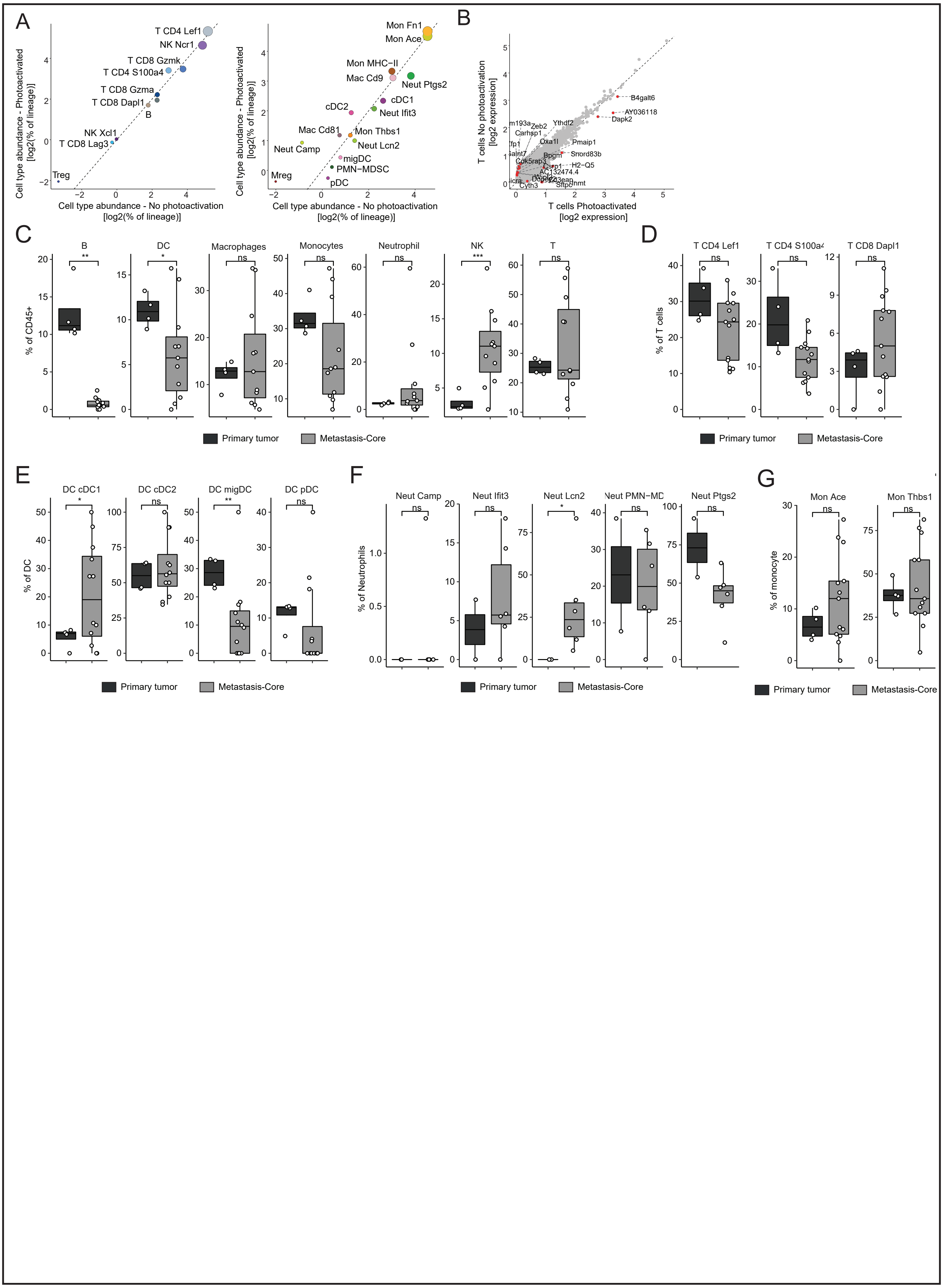

Supplementary Figure 2. Lung metastases and primary tumors exhibit divergent immune landscapes.
A. Comparison of GFP$^+$ (photoactivated) and GFP$^-$ -sorted control mouse lungs T and NK (left) or myeloid (right) cells subpopulation cell fractions (point size corresponds to mean of two samples).
B. Comparison of gene expression (log2 normalized) of photoactivated or not photoactivated T cells from control mouse lungs.
C. Fractions of indicated cell types out of total CD45$^+$ cells.
D. Fractions of indicated T cell subtypes from total T cells.
E. Fractions of indicated DC subtypes from total DC cells.
F. Fractions of indicated neutrophil subtypes from total neutrophils.
G. Fractions of indicated monocyte subtypes from total monocytes.

Two-tailed Student's t-test was used. In boxplots, the center line represents the median, the box limits denote the 25th to the 75th percentile, and the whiskers represent the minimum and maximum values. Differentially expressed genes (DEGs) are colored in red and leading DEGs are labeled.
