# Supplementary material for: Spatial and Temporal Mapping of Breast Cancer Lung Metastases Identify TREM2 Macrophages as Regulators of the Metastatic Boundary
Source: Cancer Discov. Author manuscript; Available in PMC 2025 Jul 22. (PMC7617931; doi:10.1158/2159-8290.CD-23-0299)
Supplement: Fig. s3 [file EMS206810-supplement-Fig__s3.pdf]

Supplementary Figure 3

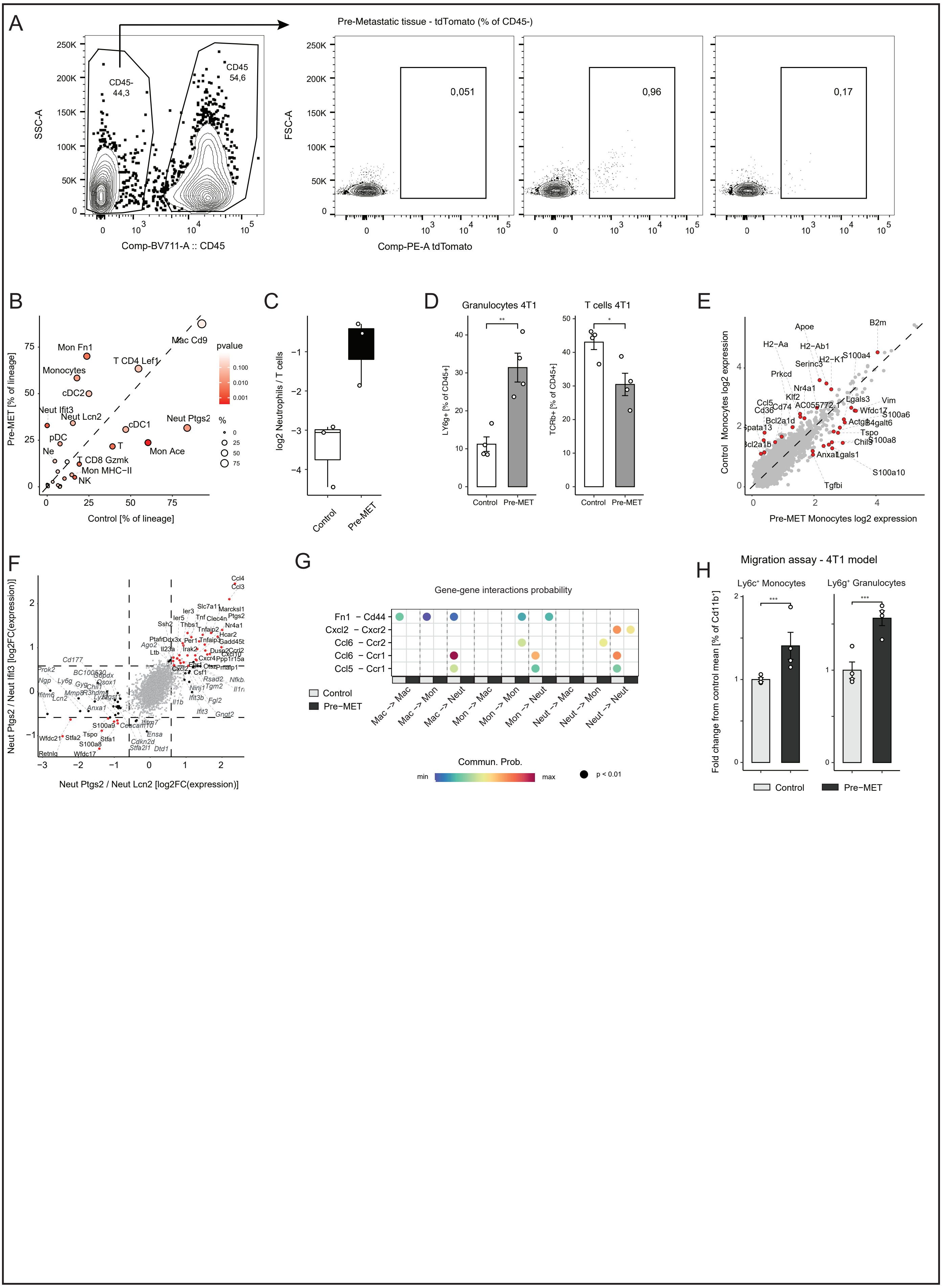

Supplementary Figure 3. The pre-metastatic lung microenvironment is characterized by activation of monocytes and neutrophils.

A. Flow cytometry analysis of EO771-tdTomato cells in Pre-MET lung tissues. Quantification of tdTomato^{+} (PE) cancer cells as percent from CD45^{-}. Each Pre-MET plot is from a different mouse examined.

B. Fractions of cells belonging to different immune lineages (from total) or subtypes (from their respective lineage), averaged over control (x axis) or Pre-MET (y axis) samples. Size indicates the average of x and y. Color gradient depicts p-value of two-sided T-test between x and y, accounting for sample variation.

C. The log2 ratio of neutrophils and T cells per sample. In boxplots, the center line represents the median, the box limits denote the 25th to the 75th percentile, and the whiskers represent the minimum and maximum values.

D. Quantification of T cells (CD45^{+}TCRb^{+}) and granulocytes (CD45^{+}CD11b^{+}Ly6g^{+}) in lungs of Balb/c mice control or Pre-MET (3 weeks after orthotopic 4T1 tumor cells injection).

E. Comparison between gene expression (log2 normalized) of monocytes from control and Pre-MET samples.

F. Comparison of gene expression fold change (FC) between neutrophil subtypes. Differentially expressed genes (DEGs) are colored in red and leading DEGs are labeled.

G. Ligand-receptor pairs probability of interaction in control and Pre-MET cells (Methods).

H. Quantification of migrated Ly6c^{+} monocytes and Ly6g^{+} granulocytes toward supernatant from normal or pre-MET lung secreted factors in the 4T1 model (presented as log2 fold-change from the normal mean, error bars denote SE).
