# Supplementary material for: Spatial and Temporal Mapping of Breast Cancer Lung Metastases Identify TREM2 Macrophages as Regulators of the Metastatic Boundary
Source: Cancer Discov. Author manuscript; Available in PMC 2025 Jul 22. (PMC7617931; doi:10.1158/2159-8290.CD-23-0299)
Supplement: Fig. s4 [file EMS206810-supplement-Fig__s4.pdf]

Supplementary Figure 4

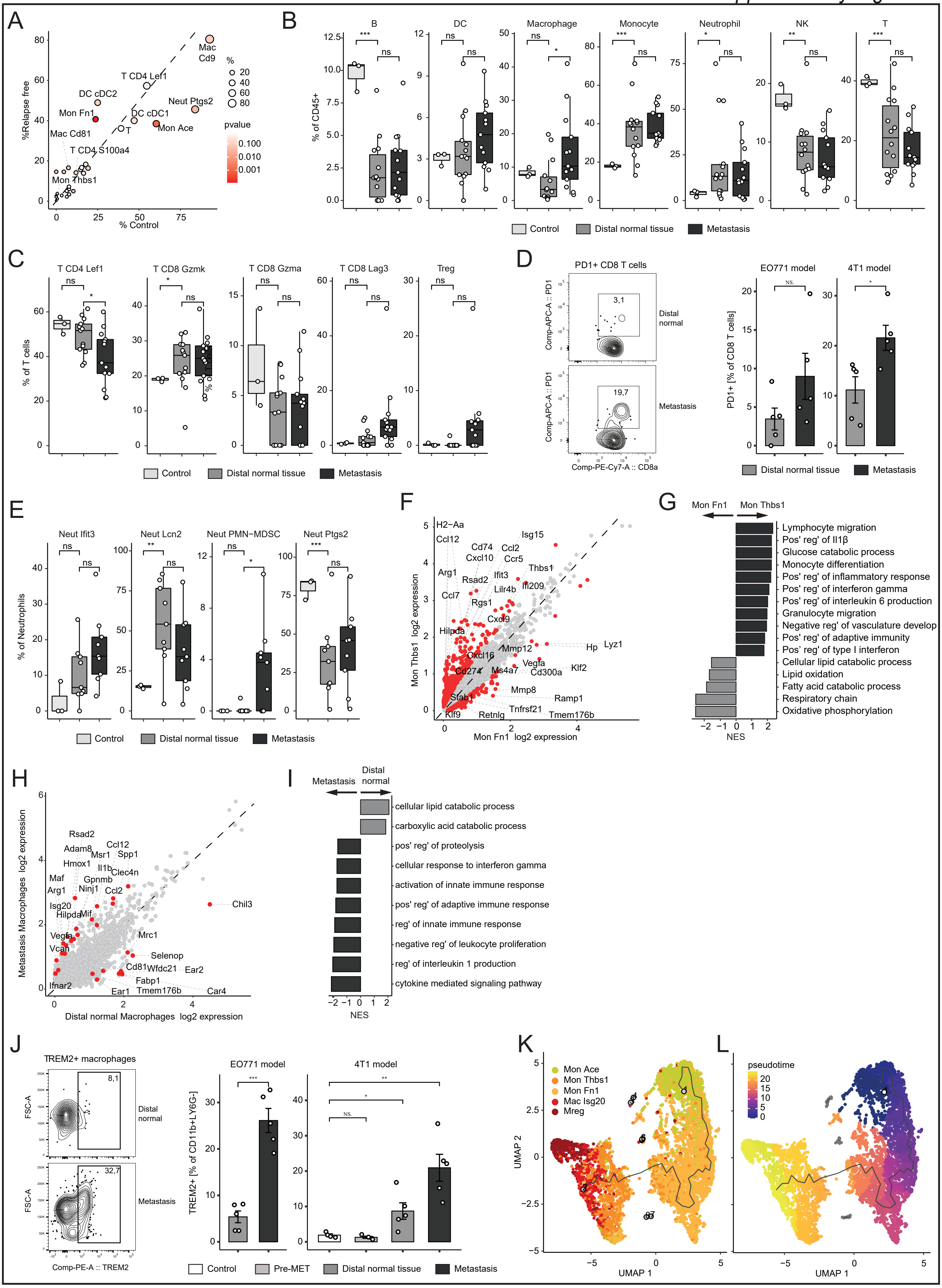

Supplementary Figure 4. Progression to lung metastasis is associated with infiltration by unconventional immune cell subtypes.

A. Fractions of cells belonging to different immune lineages (from total) or subtypes (from their respective lineage), averaged over control (x axis) or relapse-free (y axis) samples. Size indicates the average of x and y. Color depicts p-value of two-sided t-test between x and y, accounting for sample variation.

B. Fractions of indicated cell types out of total $CD45^+$ cells.

C. Fractions of indicated T cell subtypes from total T cells.

D. Representative flow cytometry plots and quantification of $PD1^+$ CD8 T cells in EO771 or 4T1 distal normal and metastatic lung tissues. Gating was performed on live, $CD45^+$, $TCRb^+$, $CD8^+$ cells.

E. Fractions of indicated neutrophil subtypes from total neutrophils.

F. Comparison of monocyte subtypes gene expression (log2 normalized).

G. Enriched gene ontology terms in monocyte subtypes.

H. Comparison between gene expression (log2 normalized) of macrophages from distal normal and metastasis tissue samples.

I. Enriched gene ontology terms in macrophages distal normal and metastasis tissue samples. Normalized GO term enrichment score (NES) is shown on x-axis. For all terms, padj < 0.05.

J. Representative flow cytometry plots and quantification of $TREM2^+$ macrophages in EO771 distal normal and metastatic lung tissues (left), or 4T1 control, Pre-MET, distal normal, and metastatic lung tissues (right). Gating was performed on live, $CD45^+$, $CD11b^+$ cells.

K. Monocyte-macrophage pseudotime trajectory (Monocle 3, Methods) projected on UMAP (Mon Ace defined as the source, cells colored by subpopulation).

L. Pseudotime analysis of (G), cells colored by pseudotime.

Two-tailed Student's t-test was used. In boxplots, the center line represents the median, the box limits denote the 25th to the 75th percentile, and the whiskers represent the minimum and maximum values. Differentially expressed genes (DEGs) are colored in red and leading DEGs are labeled. Normalized GO term enrichment score (NES) is shown on x-axis. For all terms, padj < 0.05.
