# Supplementary material for: Spatial and Temporal Mapping of Breast Cancer Lung Metastases Identify TREM2 Macrophages as Regulators of the Metastatic Boundary
Source: Cancer Discov. Author manuscript; Available in PMC 2025 Jul 22. (PMC7617931; doi:10.1158/2159-8290.CD-23-0299)
Supplement: Fig. s5 [file EMS206810-supplement-Fig__s5.pdf]

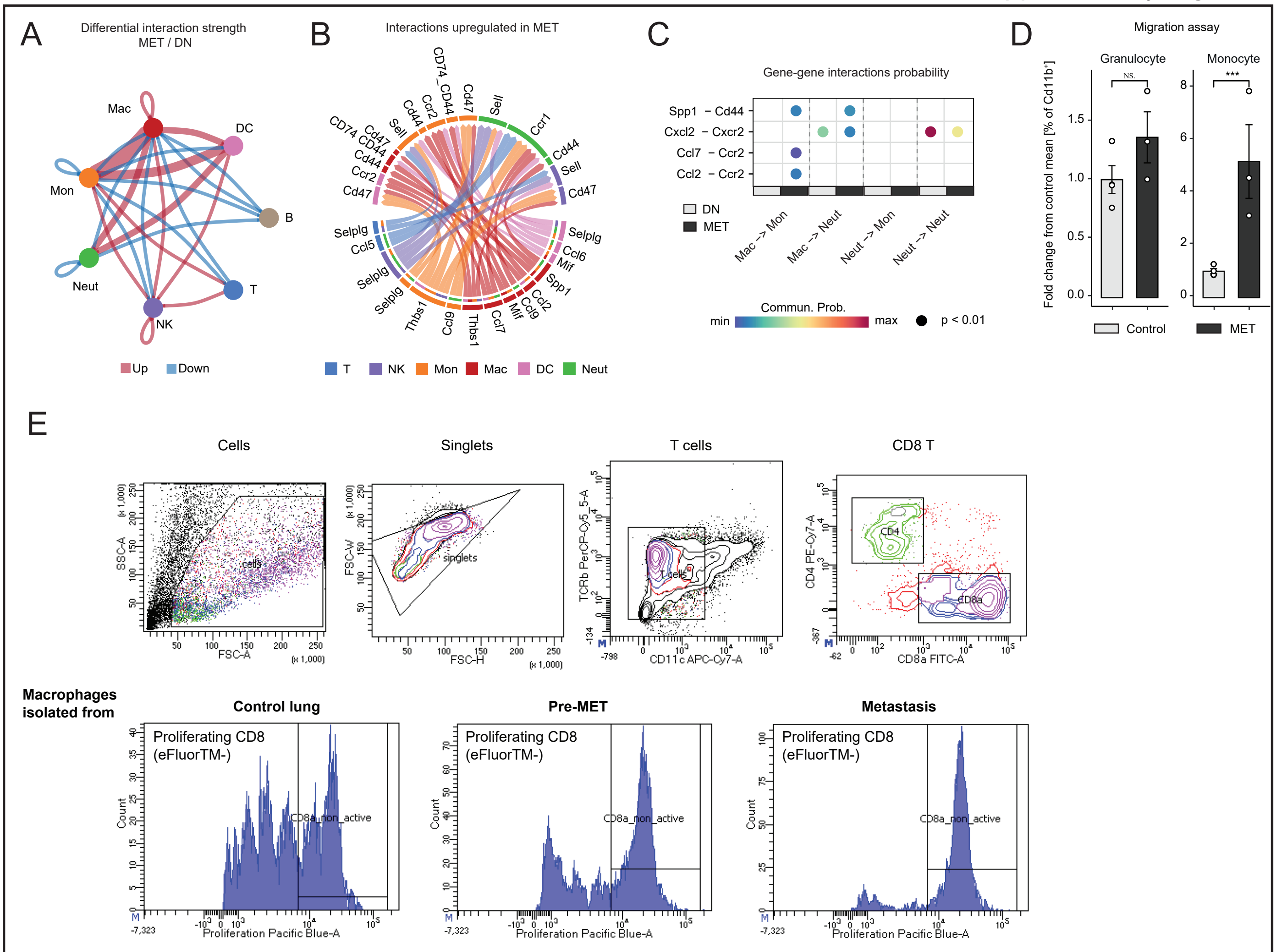
A
Differential interaction strength
MET / DN
Mac
DC
Mon
B
Neut
T
NK
Up
Down
B
Interactions upregulated in MET
T
NK
Mon
Mac
DC
Neut
C
Gene-gene interactions probability
Spp1 - Cd44
Cxcl2 - Cxcr2
Ccl7 - Ccr2
Ccl2 - Ccr2
DN
MET
Mac -> Mon
Mac -> Neut
Neut -> Mon
Neut -> Neut
Commun. Prob.
min
max
p < 0.01
D
Migration assay
Granulocyte
Monocyte
Fold change from control mean [% of Cd11b+]
Control
MET
E
Cells
Singlets
T cells
CD8 T
SSC-A
FSC-A
FSC-W
FSC-H
TCRb PerCP-Cy5_5-A
CD11c APC-Cy7-A
CD4 PE-Cy7-A
CD8a FITC-A
Macrophages isolated from
Control lung
Pre-MET
Metastasis
Proliferating CD8 (eFluorTM-)
CD8a_non_active
Count
Proliferation Pacific Blue-A

Supplementary Figure 5. Cell-cell interactions among immune cells vary within metastatic lung tissues.

A. CellChat analysis (Methods) of differential interaction strength between cell types in metastatic and distal normal tissues, based on ligand-receptor gene expression. Up depicts higher in metastasis.

B. Upregulated ligand-receptor interactions in metastasis compared to distal normal tissue, per cell type (Methods).

C. Ligand-receptor pairs probability of interaction in metastasis and distal normal tissues (Methods).

D. Ex vivo cell migration assay. Cells were isolated from bone marrow of normal mice. The lung non-cellular supernatant was produced from normal or metastatic lung tissues. Quantification of migrated Ly6C^{+} monocytes or Ly6G^{+} granulocytes toward supernatants from control or metastatic lungs, presented as fold-change from control mean, error bars denote SE.

E. Gating scheme for quantification of proliferating CD8 T cells (eFluorTM 450 diluted).
