# Supplementary material for: Spatial and Temporal Mapping of Breast Cancer Lung Metastases Identify TREM2 Macrophages as Regulators of the Metastatic Boundary
Source: Cancer Discov. Author manuscript; Available in PMC 2025 Jul 22. (PMC7617931; doi:10.1158/2159-8290.CD-23-0299)
Supplement: Fig. s6 [file EMS206810-supplement-Fig__s6.pdf]

Supplementary Figure 6

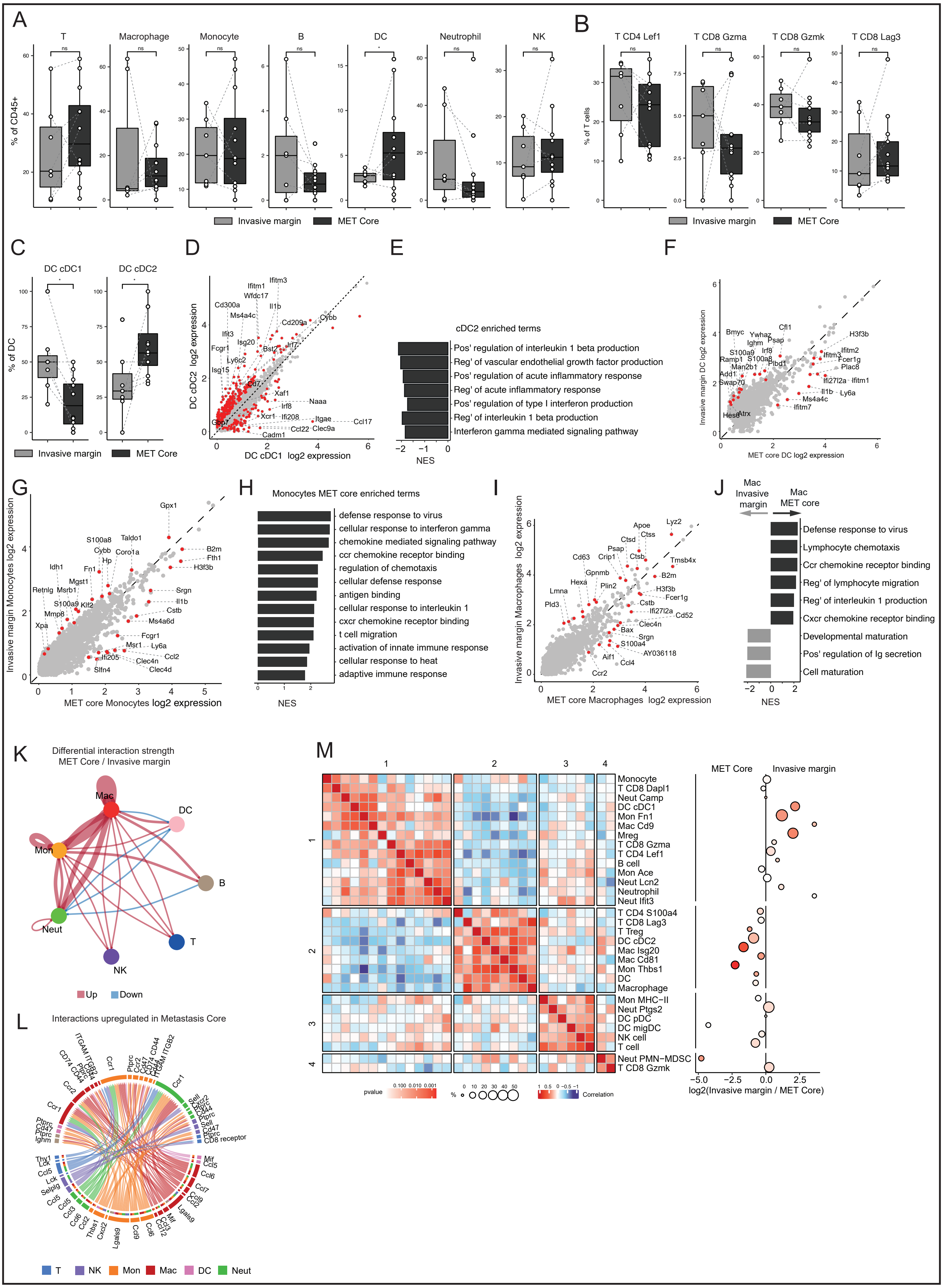

Supplementary Figure 6. The metastatic core and invasive margin are populated by distinct macrophage subpopulations.

A. Fractions of indicated cell types out of total $CD45^{+}$ cells.

B. Fractions of indicated T cell subtypes from total T cells.

C. Fractions of indicated DC subtypes from total DC cells.

D. Comparison of DC subtypes gene expression (log2 normalized).

E. Enriched gene ontology terms in cDC2 compared to cDC1.

F. Comparison between gene expression (log2 normalized) of DCs from metastases core and invasive margin.

G. Comparison between gene expression (log2 normalized) of monocytes from metastases core and invasive margin.

H. Enriched gene ontology terms in monocytes from metastasis core compared to invasive margin.

I. Comparison between gene expression (log2 normalized) of macrophages from metastases core and invasive margin.

J. Enriched gene ontology terms in macrophages from metastasis core compared to invasive margin.

K. CellChat analysis (Methods) of differential interaction strength between cell types in metastasis core and invasive margin, based on ligand-receptor gene expression. Up depicts higher in metastasis core.

L. Upregulated ligand-receptor interactions in metastasis core compared to invasive margin, per cell type (Methods).

M. Cellular module analysis. Pairwise Spearman correlation of cell type and subpopulation fraction across cells from metastasis invasive margin and core (left; Color gradient represents Spearman correlation). Consensus hierarchical clustering into four cell modules. Enrichment of each cell type between metastasis invasive margin and core (right). Size indicates mean percentage of cells in all samples; Color gradient represents the p-value of Student's t-test.

Two-tailed paired t-test was used. In boxplots, the center line represents the median, the box limits denote the 25th to the 75th percentile, and the whiskers represent the minimum and maximum values. Differentially expressed genes (DEGs) are colored in red and leading DEGs are labeled. Normalized GO term enrichment score (NES) is shown on x-axis. For all terms, $padj < 0.05$.
