# Supplementary material for: Spatial and Temporal Mapping of Breast Cancer Lung Metastases Identify TREM2 Macrophages as Regulators of the Metastatic Boundary
Source: Cancer Discov. Author manuscript; Available in PMC 2025 Jul 22. (PMC7617931; doi:10.1158/2159-8290.CD-23-0299)
Supplement: Fig. s7 [file EMS206810-supplement-Fig__s7.pdf]

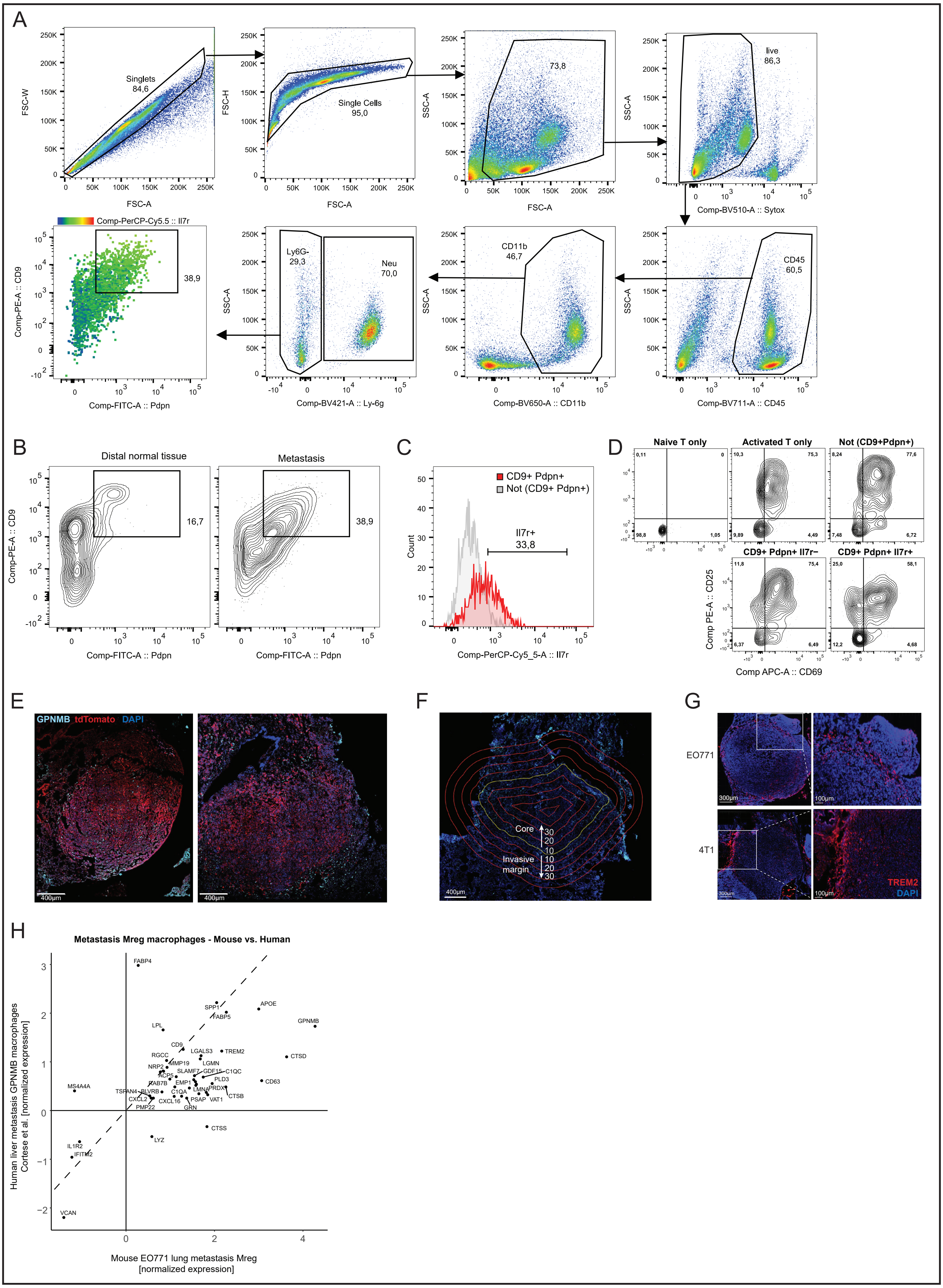
A
Singlets
84,6
FSC-W
FSC-A
Single Cells
95,0
FSC-H
73,8
SSC-A
live
86,3
Comp-BV510-A :: Sytox
Comp-PerCP-Cy5.5 :: Il7r
Comp-PE-A :: CD9
38,9
Comp-FITC-A :: Pdpn
Ly6G-
29,3
Neu
70,0
Comp-BV421-A :: Ly-6g
CD11b
46,7
Comp-BV650-A :: CD11b
CD45
60,5
Comp-BV711-A :: CD45
B
Distal normal tissue
Metastasis
16,7
38,9
C
CD9+ Pdpn+
Not (CD9+ Pdpn+)
Il7r+
33,8
Count
Comp-PerCP-Cy5_5-A :: Il7r
D
Naive T only
Activated T only
Not (CD9+Pdpn+)
CD9+ Pdpn+ Il7r−
CD9+ Pdpn+ Il7r+
Comp PE-A :: CD25
Comp APC-A :: CD69
E
GPNMB tdTomato DAPI
400µm
F
Core
Invasive margin
30
20
10
G
EO771
4T1
300µm
100µm
TREM2
DAPI
H
Metastasis Mreg macrophages - Mouse vs. Human
Human liver metastasis GPNMB macrophages Cortese et al. [normalized expression]
Mouse EO771 lung metastasis Mreg [normalized expression]
FABP4
SPP1
APOE
FABP5
GPNMB
LPL
CD9
LGALS3
TREM2
RGCC
CTSD
NRP2
MMP19
LGMN
SLAMF7
GDF15
C1QC
ACP5
FABP5
EMP1
PLD3
CD63
MS4A4A
TSPAN4
BLVRB
C1QA
LMNA
PRDX1
CTSB
CXCL2
CXCL16
PSAP
VAT1
PMP22
GRN
CTSS
LYZ
IL1R2
IFITM2
VCAN

Supplementary Figure 7. The metastatic invasive margin is populated by suppressive TREM2 macrophages

A. Gating strategy for $CD45^+$ $CD11b^+$ $Ly6G^-$ $CD9^+$ $PDPN^+$ cells. On the last plot the color heatmap indicates IL7R expression.

B. Comparison of $CD9^+$ $PDPN^+$ cells between representative distal normal and metastasis tissues.

C. IL7R expression level histogram for the $CD9^+$ $PDPN^+$ double positive population (red) and the inverse gate (grey).

D. Gating strategy for activated T cells, with representative flow cytometry plots for each condition. Gating was set for $CD45^+$, $TCRb^+$, $CD8^+$, and activated cells were $CD25^+$ $CD69^+$ double positive.

E. Representative immuno-fluorescence imaging of EO771 breast cancer lung metastasis, stained for GPNMB (cyan). Tumor cells are shown in red (tdTomato), nuclei in blue (Dapi).

F. An example of the quantification of cell presence in spatial regions of metastases. The metastasis boundary was determined by cell (Dapi) density and drawn manually (Yellow line). This boundary was used to determine decile spatial areas inward to the metastasis center or outward to the surrounding invasive margin or stroma (Methods).

G. Representative immuno-fluorescence imaging of EO771 and 4T1 breast cancer lung metastasis, stained for TREM2 (red) and nuclei (blue, Dapi).

H. Comparison between gene expression enrichment (log2 normalized) of Mreg macrophages in mouse EO771 lung metastasis (this study) and GPNMB+ macrophages in liver metastasis (61).
