# Supplementary material for: Spatial and Temporal Mapping of Breast Cancer Lung Metastases Identify TREM2 Macrophages as Regulators of the Metastatic Boundary
Source: Cancer Discov. Author manuscript; Available in PMC 2025 Jul 22. (PMC7617931; doi:10.1158/2159-8290.CD-23-0299)
Supplement: Fig. s8 [file EMS206810-supplement-Fig__s8.pdf]

Supplementary Figure 8

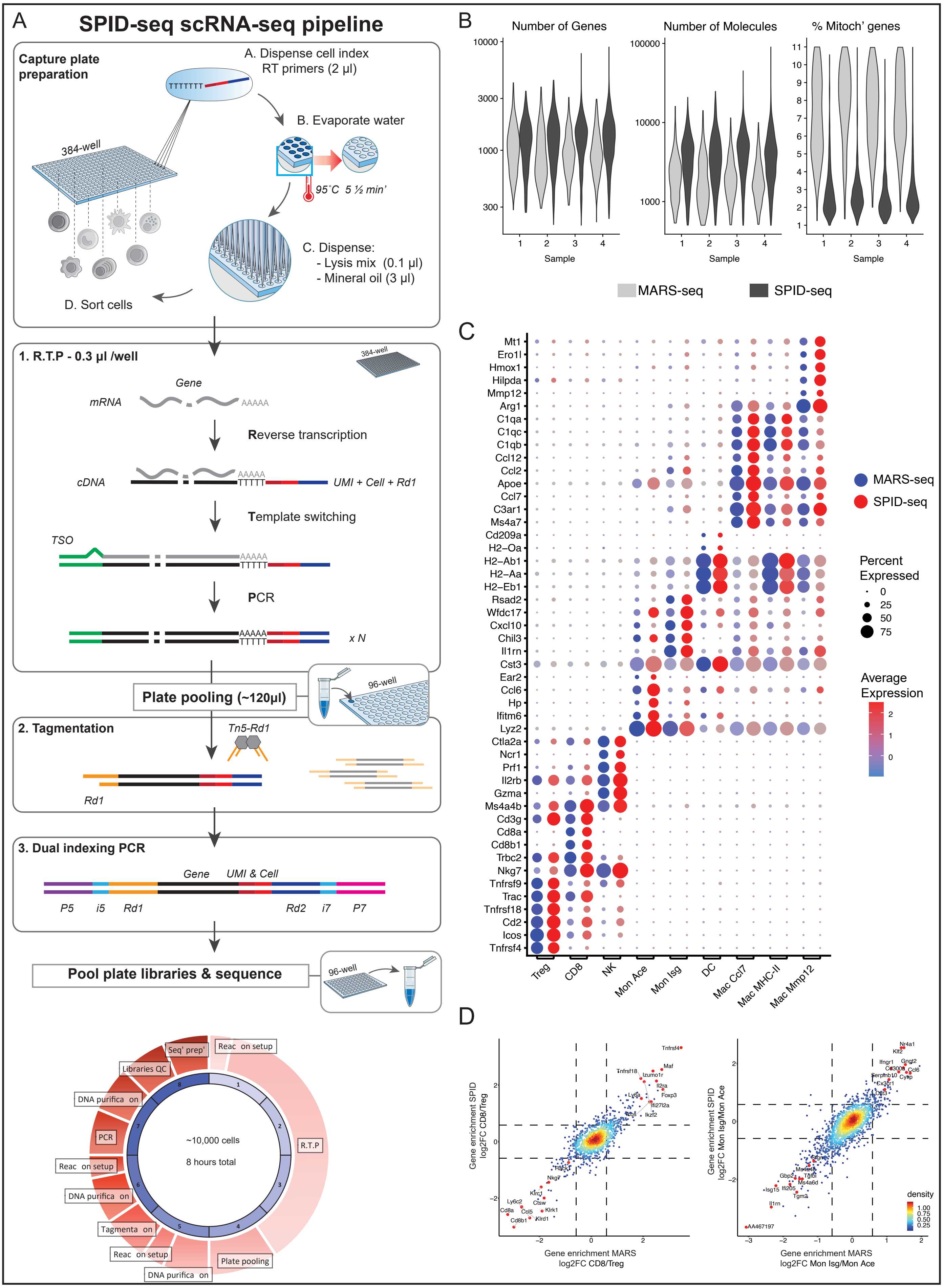

Supplementary Figure 8. The SPID-seq method for plate-based scRNA-seq.

A. Schematic highlights of the key molecular steps in SPID-seq, and a timeline for processing of ~10,000 cells (when done manually).

B. MC38 tumor-infiltrating immune cells ($CD45^+$) from four mice were subjected to scRNA-seq by MARS-seq (73) or SPID-seq. The violin plots represent the distribution of the number of genes, unique molecular identifiers (UMI), and percent of mitochondrial gene expression (indicating low-quality cells) per sample.

C. Comparison of the expression of differentially expressed genes that define immune subpopulations between MARS-seq and SPID-seq.

D. Comparison of gene enrichment between four defined subpopulations in MARS-seq (x-axis) and SPID-seq (y-axis).
